# Metagenomic Comparison of Bat Colony Resistomes Across Anthropogenic and Pristine Habitats

**DOI:** 10.3390/antibiotics15010051

**Published:** 2026-01-03

**Authors:** Julio David Soto-López, Omar Velásquez-González, Manuel A. Barrios-Izás, Moncef Belhassen-García, Juan Luis Muñoz-Bellido, Pedro Fernández-Soto, Antonio Muro

**Affiliations:** 1 Infectious and Tropical Diseases Research Group (e-INTRO), Biomedical Research Institute of Salamanca-Research Centre for Tropical Diseases (IBSAL-CIETUS), Faculty of Pharmacy, University of Salamanca, 37008 Salamanca, Spain; jdjuliosoto@usal.es; 2 Computational Chemistry Unit, School of Chemistry, University of San Carlos Guatemala, Guatemala City 01012, Guatemala; velasquez_omar@profesor.usac.edu.gt; 3 Research Institute, University Center of Zacapa, University of San Carlos of Guatemala, Zacapa 01019, Guatemala; manuelbarrios@cunzac.edu.gt; 4 Internal Medicine Service, Infectious Diseases Unit, HUS, IBSAL, e-INTRO, CIETUS, University of Salamanca, 37007 Salamanca, Spain; belhassen@usal.es (M.B.-G.); jlmubel@usal.es (J.L.M.-B.)

**Keywords:** antimicrobial, bats, resistance, microbial, shotgun metagenomics

## Abstract

Background/Objectives: The mammalian microbiota constitutes a reservoir of antimicrobial resistance genes (ARGs), which can be shaped by environmental and anthropogenic factors. Although bat-associated bacteria have been reported to harbor diverse ARGs globally, the ecological and evolutionary determinants driving this diversity remain unclear. Methods: To characterize ARG diversity in wildlife exposed to contrasting levels of human influence, we analyzed homologs of resistance mechanisms from the Comprehensive Antibiotic Resistance Database in shotgun metagenomes of bat guano. Samples were collected from a colony exposed to continuous anthropogenic activity in Spain (Salamanca) and from a wild, non-impacted bat community in China (Guangdong). Metagenomic analyses revealed marked differences in taxonomic and resistome composition between sites. Results: Salamanca samples contained numerous hospital-associated genera (e.g., *Mycobacterium*, *Staphylococcus*, *Corynebacterium*), while Guangdong was dominated by *Lactococcus*, *Aeromonas*, and *Stenotrophomonas*. β-lactamases and MurA transferase homologs were the most abundant ARGs in both datasets, yet Salamanca exhibited higher richness and functional diversity (median Shannon index = 1.5; Simpson = 0.8) than Guangdong (Shannon = 1.1; Simpson = 0.66). Salamanca also showed enrichment of clinically relevant ARGs, including *qacG*, *emrR*, *bacA*, and *acrB*, conferring resistance to antibiotics critical for human medicine. In contrast, Guangdong exhibited a more restricted resistome dominated by β-lactamase and MurA homologs. Beta diversity analysis confirmed significant compositional differences between resistomes (PERMANOVA, R^2^ = 0.019, F = 1.33, *p* = 0.001), indicating ecological rather than stochastic structuring. Conclusions: These findings suggest that anthropogenic exposure enhances the diversity and evenness of resistance mechanisms within bat-associated microbiomes, potentially increasing their role as reservoirs of antimicrobial resistance.

## 1. Introduction

Microorganisms are the most abundant and diverse life forms on Earth, occupying virtually every conceivable metabolic niche [1,2,3,4]. The microbial communities colonizing the mammalian gut exert profound influences on host physiology and health [5], contributing to host adaptation to new ecological niches through the production of bioactive metabolic compounds [6]. While gut microbial composition and abundance are shaped by various stochastic external factors, dietary inputs [7] and host genetic factors [5] have been described as major drivers of microbial community dynamics.

Mammalian gut microbiota comprise eukaryotes, archaea, viruses, and bacteria that are essential for host well-being and also act as reservoirs of antibiotic-resistant bacteria and their associated antibiotic resistance genes (ARGs) [8,9], collectively forming the resistome. Positive selection of ARGs can be promoted by nutrient availability, temperature, pH, and exposure to natural or anthropogenic chemicals [10,11]. Furthermore, environmental factors and interspecific bacterial interactions facilitate horizontal gene transfer (HGT) of ARGs via transformation, conjugation, or transduction [12,13]. Both HGT and environmental selective pressures play pivotal roles not only in the acquisition of ARGs but also in their diversification within natural environments [14].

Bat intestinal microbiota has been shown to harbor a range of bacterial taxa that include opportunistic and clinically relevant pathogens, such as *Escherichia*, *Salmonella*, *Campylobacter*, *Staphylococcus*, *Enterococcus*, *Mycobacterium*, and *Aeromonas* species [15,16,17]. Several of these genera are known to carry antimicrobial resistance mechanisms, including β-lactamases, multidrug efflux pumps, target modification enzymes, and resistance determinants against aminoglycosides, fluoroquinolones, and disinfectants [18,19]. While many of these bacteria may behave as commensals within bat hosts, they can become pathogenic under altered ecological or immunological conditions, posing risks to bat health and acting as reservoirs of resistance genes with zoonotic potential [19,20,21].

Transmission of antimicrobial-resistant bacteria and resistance genes between bats and humans can occur through multiple pathways, including environmental contamination of roosting sites, guano accumulation, water and soil interfaces, indirect contact via domestic animals, and increasing human encroachment into bat habitats through tourism, urbanization, and wildlife management practices [15,22]. Importantly, such transmission dynamics are bidirectional: bats may acquire resistant microorganisms from human-impacted environments, hospitals, or agricultural settings, while humans may be exposed to bat-associated bacteria and resistance genes through occupational or recreational contact [23,24]. Owing to their high species diversity and frequent contact with humans [25,26], bat-associated microbial communities increasingly harbor bacteria resistant to multiple antibiotics [27,28]. Given the potential risk of transmitting resistant bacteria to human populations, understanding the ecological and evolutionary dynamics that maintain or transmit ARGs in these systems is essential.

The resistome in bacterial communities is commonly investigated using tools such as BLAST or the Resistance Gene Identifier (RGI) [29,30,31], which compare genetic sequences derived from shotgun metagenomic data, the most widely used approach for studying and classifying microorganisms in complex environments [32,33], against curated ARG databases [30,34,35,36]. However, these databases are predominantly constructed from ARGs identified in human-associated bacteria. Because these searches rely on sequence similarity to known ARGs, they may yield a biased underestimation of ARG diversity, resulting in inflated false-negative rates. Bacterial lineages in bats have often evolved in isolation under distinct selective pressures, and many ARGs in their microbiomes may represent distant homologs, novel functional variants, or entirely undescribed resistance determinants [37]. Additionally, cross-contamination, particularly in sites receiving hundreds of visitors, as in our focal Spanish location, may lead to false positives.

To overcome these limitations, an alternative approach involves the study of sequence coevolution. This strategy is grounded in the observation that the conserved function of a protein family imposes strict constraints on sequence variability, often resulting in conserved structural features among all family members [38]. To maintain energetically favorable molecular interactions, amino acid residues in spatial proximity may coevolve in a coordinated manner across the family [31,39,40]. Recognition of homologous proteins based solely on sequence similarity does not guarantee functional equivalence, as homology can be confounded with analogy or convergent evolution, potentially leading to erroneous functional annotations. Incorporating phylogenetic analyses can improve the accuracy of both functional and taxonomic assignments and can reveal lineage-specific expansions associated with ecological adaptations [41,42].

In northwestern Spain, a 19th-century railway line (abandoned for more than four decades) has become a refuge for several bat species. The unique topography of this region creates diverse microclimates that favor the presence of bat species such as *Rhinolophus hipposideros*, *R. ferrumequinum*, *R. euryale*, *Myotis blythii*, *M. myotis*, *M. emarginatus* and *Miniopterus schreibersii*, what make it part of the Natura 2000 network. The recent development of tourism along the “Camino del Hierro” tunnels has coincided with a gradual decline in bat populations and increased, sustained human–bat contact [43,44].

According to park staff, bat guano is periodically removed manually using shovels, and chemical disinfectants are applied to the tunnel floors to minimize microbial growth. These management practices likely alter the natural microbial composition of the site and represent an additional, recurrent anthropogenic pressure on the resident bat colonies. In a recent publication, we reported marked differences in the relative microbial composition, metabolic pathways and virulence factors, across samples from fresh and desiccated bat guano. The most abundant bacterial genera (from a human health importance perspective) are *Bacillus*, *Burkholderia*, *Lactobacillus*, *Pseudomonas*, *Salmonella*, and *Streptococcus* [45]. The presence of these taxa in the tunnels poses a potential risk not only to visitors but also to park staff who are regularly exposed to bat guano, particularly due to the presence of species associated with various human viral and bacterial diseases.

Overall, our findings highlight an underrecognized and unmonitored risk of pathogen transmission for both visitors and personnel at this tourist site, underscoring the need for increased awareness and further investigation into the health implications of human–bat interactions in such environments.

To investigate how contrasting levels of anthropogenic pressure shape the diversity and dissemination of antibiotic resistance genes (ARGs) in wildlife reservoirs, we compared two ecologically and geographically distinct bat colonies. The Spanish site represents a system under continuous and multifaceted human disturbance (tourism), mechanical guano removal, and periodic chemical disinfection where recurrent exposure to human-associated microbiota and potential antibiotic residues may promote ARG exchange. In contrast, the Chinese site hosts a wild, non-human-impacted community, providing a near-pristine baseline to assess the natural background of resistance mechanisms in bats. This dual-site design allows a robust evaluation of whether anthropogenic activity measurably alters the resistome structure in wildlife populations.

## 2. Results

### 2.1. Bat Species and Reads Quality Control

In Salamanca, bat guano samples were obtained from *Myotis myotis* (Borkhausen, 1797), *Myotis blythii* (Tomes, 1857), *Myotis* sp., *Rhinolophus ferrumequinum* (Schreber, 1774), *Miniopterus schreibersii* (Kuhl, 1817), and *Miniopterus natalensis* (A. Smith, 1834). These species are typical cave-roosting bats with overlapping dietary niches dominated by insect prey.

In contrast, samples from Guangdong were derived from bats captured in Yan-yan Cave (Huizhou City, China) and included *Myotis pilosus* (Wagner, 1848), *Myotis chinensis* (Milne-Edwards, 1872), *Myotis altarium* (Thomas, 1911), *Myotis davidii* (Milne-Edwards, 1866), *Myotis longipes* (Dobson, 1873), *Rhinolophus pearsonii* (Horsfield, 1851), *Rhinolophus pusillus* (Temminck, 1835), *Rhinolophus siamensis* (Andersen, 1918), *Rhinolophus affinis* (Horsfield, 1823), *Rhinolophus macrotis* (Blyth, 1844), *Hypsugo pulveratus* (Blyth, 1853), *Eptesicus serotinus* (Schreber, 1774), *Pipistrellus abramus* (Temminck, 1835), *Murina huttoni* (Gray, 1842), *Murina aurata* (Hodgson, 1835), *Miniopterus pusillus* (Peters, 1867), *Pteropus fuliginosus* (Temminck, 1837) synonymous to *Pteropus lylei* (Andersen, 1908) and *Rousettus leschenaultii* (Desmarest, 1820).

Although no bat species were shared between the two sampling sites, both assemblages included representatives of the genera *Myotis*, *Rhinolophus*, and *Miniopterus*, which comprise predominantly insectivorous, cave-roosting bats with comparable ecological traits.

From the guano samples, we obtained from 19,744,592 to 43,824,959 raw reads (150 bp sequence length and 2.9 to 6.5 gigabases -Gbp-) in Salamanca and from 7,946,402 to 41,413,942 raw reads (150 bp sequence length and 1.1 to 6.2 gigabases -Gbp-) in Guangdong. After sample preprocessing, the number of reads per sample ranged from 14,328,952 to 28,221,077 reads (100–150 bp sequence length and 2 to 3.9 Gbp) in Salamanca and from 1,256,476 to 28,708,812 reads (100–150 bp sequence length and 0.1827 to 4.2 Gbp) in Guangdong [see Supplementary Material File: Sample_inf].

### 2.2. Metagenome-Assembled Genomes

Following the assembly performed, we obtained a total of 199 Metagenome-Assembled Genomes (MAGs) from the Salamanca, Spain dataset and 100 from the Guangdong, China dataset [see Supplementary Material File: MAGs_report], with completeness ranging between 90% and 100%, and contamination levels between 0% and 5%. We successfully identified 50 bacterial species and 157 bacterial genera, along with one archaeal MAG (Phylum Thermoproteota) in Salamanca, and 63 bacterial species and 88 bacterial genera in Guangdong. Among the identified species, a core set, though not exclusively composed of *Enterococcus faecalis*, *Lactococcus lactis*, *Escherichia coli*, and *Sarcina mediterraneensis,* was observed. Notable human health-relevant pathogens include *Clostridium perfringens* and *Citrobacter* spp. (Salamanca), as well as *Bacillus cereus*, *Morganella morganii*, *Providencia* spp., *Proteus* spp., and *Aeromonas* spp. (Guangdong). Lactic acid/fermentative bacteria identified include *Lactococcus garvieae* (both locations), *Latilactobacillus sakei*, *Weissella muntiaci*, and *Staphylococcus nepalensis* (Salamanca).

Several MAGs obtained from Salamanca phylogenetically cluster with reference genomes known to harbor resistance genes as annotated in the Comprehensive Antibiotic Resistance Database (CARD) (bootstrap/UFBoot ≥ 95; 1000 replicates; MFP model; bb: 1000; UFBoot: 1000).

**Figure 1:**
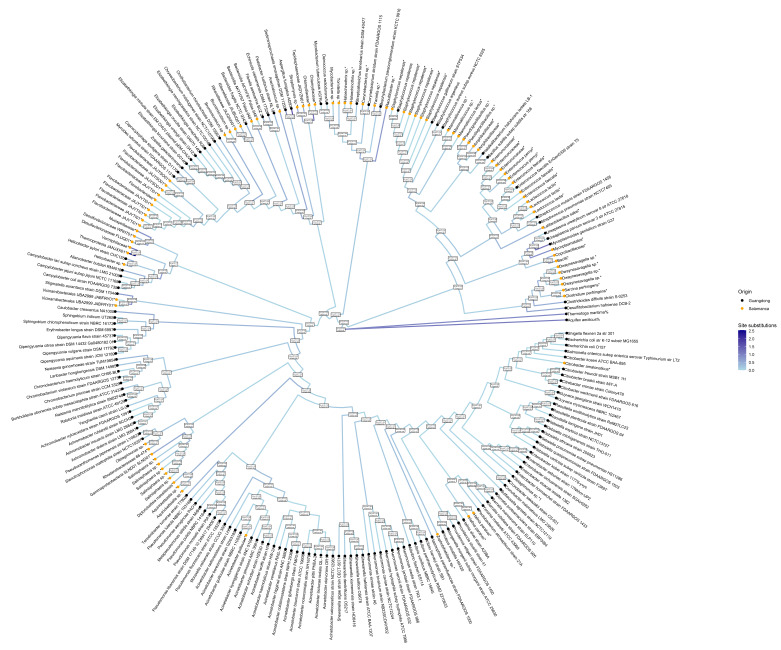
Circular phylogenetic tree of CARD reference bacterial species and Salamanca MAGs constructed using single-copy BUSCO genes. The color of the branches represents the expected number of substitutions per site under the LG + G + F evolutionary model, indicating the relative amount of genetic change between sequences. Nodes display SH-aLRT and ultrafast bootstraps (UFboot) support values, calculated with 1000 replicates using IQ-TREE under the LG+G+F model. Alignments were filtered with a 0.7 gap threshold to retain high-quality positions. Only MAGs with sufficient BUSCO markers passing the filter were included; MAGs lacking these markers were excluded from the tree. %Outer groups, * Salamanca MAG’s.

Phylogenetic analysis (IQ-TREE; LG substitution matrix with empirical amino acid frequencies + gamma-distributed rate heterogeneity; N sites = 34,780; SH-aLRT/UFBoot = 1000 replicates) reveals that multiple MAGs assembled from Salamanca samples (*highlighted in orange) cluster within clades alongside previously reported reference genomes carrying resistance determinants documented in CARD (Figure 1). Specifically, several Salamanca-derived MAGs form robustly supported clades (SH-aLRT/UFBoot ≥ 95) with reference genomes of *Enterococcus*, *Lactococcus*, *Hafnia*, *Helicobacter*, *Staphylococcus*, *Streptococcus*, *Stenotrophomonas*, *Acinetobacter*, *Clostridioides*, *Mycoplasmoides*, *Mycobacterium*, and *Bacillus*, all of which carry resistance genes reported in CARD. [see Supplementary Material File: Homologs_Sala].

### 2.3. Antimicrobial Resistance Genes

**Figure 2:**
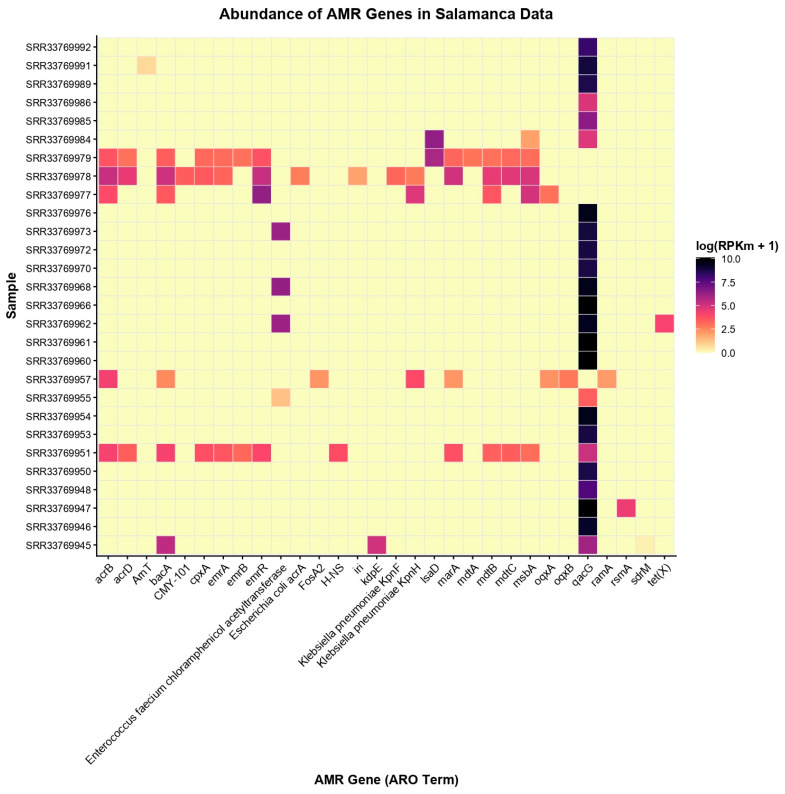
Heatmap showing the distribution of antimicrobial resistance (AMR) genes across samples from Salamanca, Spain. Sample codes correspond to individual metagenomic datasets (SRA accession numbers). Colors represent the normalized abundance of each gene as log(RPKm + 1). Rows correspond to samples and columns to AMR genes (ARO terms). Only genes that were detected in contigs, MAGs, and raw reads are included. Genes were filtered for quality: contigs with “Strict” or “Perfect” hits, and raw reads with ≥80% coverage.

Several antimicrobial resistance (AMR) gene families, including *Enterococcus faecium chloramphenicol acetyltransferase*, *emrR*, *bacA*, and *acrB*, were consistently detected across multiple samples, indicating a shared core resistome within the Salamanca colony (Figure 2). Notably, some samples (SRR33769979, SRR33769978, SRR33769977, SRR33769957, and SRR33769951) exhibited more complex and diverse AMR profiles, whereas others showed a more restricted gene repertoire, reflecting heterogeneity in resistance gene distribution among guano-associated microbial communities.

The predominance of genes such as *qacG*, which confers tolerance to quaternary ammonium compounds, suggests selective pressure from disinfectants commonly associated with anthropogenic environments. Overall, the heatmap highlights both a conserved resistome core and sample-specific enrichment patterns, consistent with microbial communities inhabiting aged and desiccated bat guano accumulated on cave floors [45]. Sample identifiers (e.g., SRR33769979) correspond to individual metagenomic datasets retrieved from the NCBI Sequence Read Archive, each representing an independent guano-derived metagenome from the Salamanca bat colony (see Supplementary Table S1 for detailed metadata).

**Figure 3:**
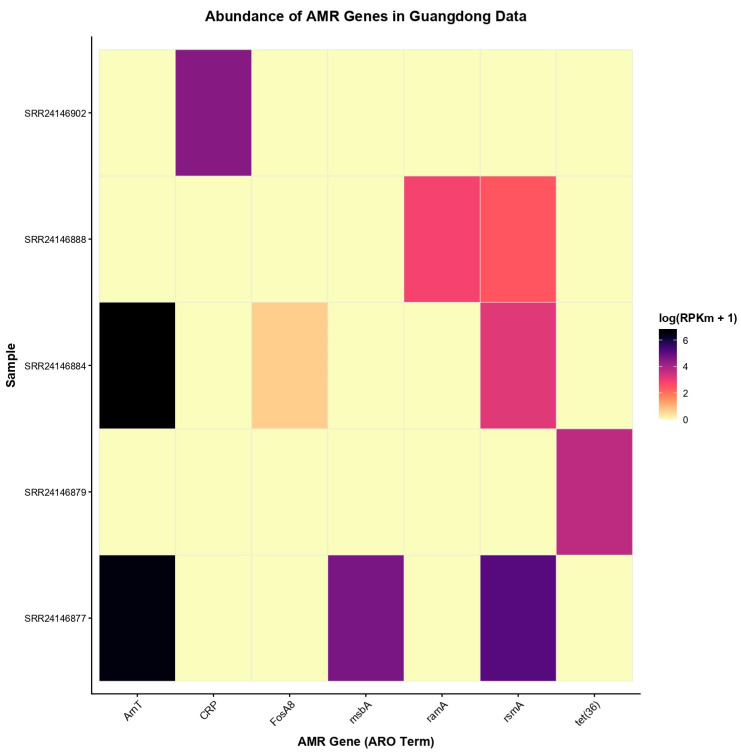
Heatmap showing the distribution of antimicrobial resistance (AMR) genes across samples from Guangdong, China. Sample codes correspond to individual metagenomic datasets (SRA accession numbers). Colors represent the normalized abundance of each gene as log(RPKm + 1). Rows correspond to samples and columns to AMR genes (ARO terms). Only genes that were detected in contigs, MAGs, and raw reads are included. Genes were filtered for quality: contigs with “Strict” or “Perfect” hits, and raw reads with ≥80% coverage.

In contrast, only five of the 36 analyzed samples from Guangdong contained detectable antimicrobial resistance (AMR) genes (Figure 3), indicating a markedly lower prevalence of resistance determinants in this site. The most abundant genes identified in these samples were *AmT*, *CRP*, *msbA*, and *rsmA*, with *rsmA* and *AmT* being the most frequently detected, each present in at least two samples. These genes are primarily associated with regulatory functions and intrinsic resistance mechanisms, such as membrane transport and stress response, rather than with acquired resistance to clinically critical antibiotics.

**Figure 4:**
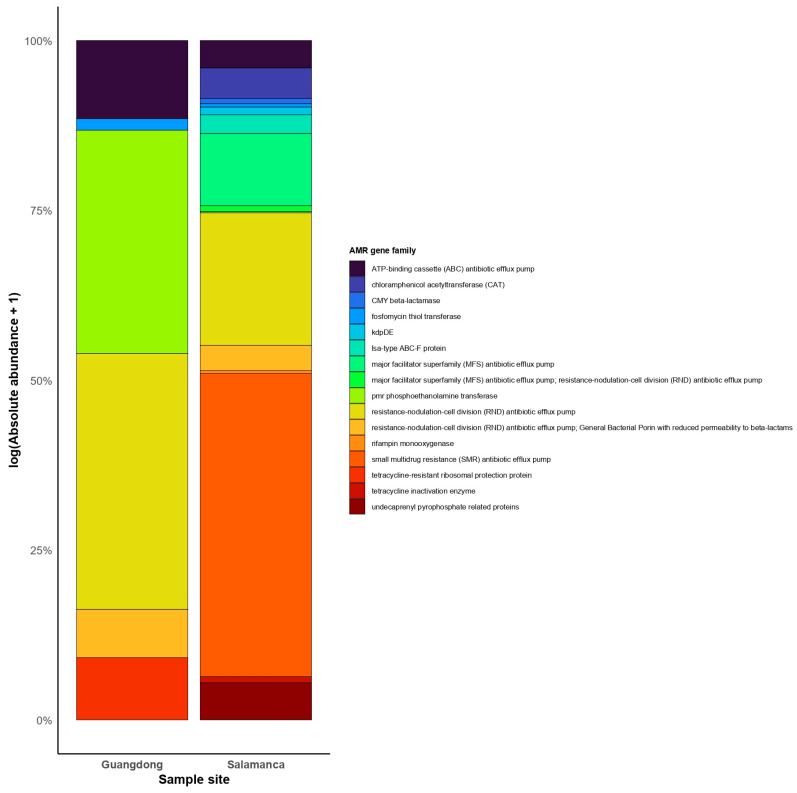
Comparison of AMR gene composition between Salamanca and Guangdong. Bars represent the cumulative normalized abundance of AMR genes (log(RPKm + 1)) grouped by gene family. Colors indicate different AMR families. Only genes families that were detected in contigs, MAGs, and raw reads are included. Genes families were filtered for quality: contigs with “Strict” or “Perfect” hits, and raw reads with ≥80% coverage.

Antimicrobial resistance gene families reveals that the microbiota in Salamanca harbors a broader and more diverse reservoir of antimicrobial resistance genes than Guangdong (Figure 4). In Guangdong, the detected genes belong to a limited number of families, including ATP-binding cassette (ABC) efflux pumps, fosfomycin transferases, *pmr* (phosphoethanolamine transferase), and RND-type efflux pumps. These exhibit moderate abundances (2.9–15.3 log(RPKM +1)) and are detected in only a small number of samples (2–5).

In contrast, Salamanca displays greater diversity and abundance of AMR gene families (Figure 4). The most prevalent include ABC and MFS efflux pumps, CMY-type β-lactamases, chloramphenicol acetyltransferases (CAT), lsa-type ABC-F proteins, *pmr*, SMR transporters, and proteins associated with undecaprenyl pyrophosphate metabolism. Notably, certain families, such as SMR and MFS, reach substantially higher abundances (47–199 log(RPKM +1)).

Phylogenetic reconstruction using IQ-TREE revealed that homologous sequences from Salamanca (denoted by SRR identifiers) cluster monophyletically with the corresponding reference genes from the CARD database (denoted by GCF identifiers), with strong branch support (SH-aLRT ≥ 80, UFboot ≥ 95; see Supplementary Materials Figures S1–S11). Monophyletic clustering indicates that the sequences share a common ancestor and are likely orthologous, implying conservation of their resistance function. In phylogenetic trees, clades are considered monophyletic when all sequences descending from a single ancestral node are included in the same group without exclusion of related sequences. Conversely, paralogous genes would form distinct clades arising from duplication events, often exhibiting shorter branch lengths if recently duplicated, whereas more divergent homologs may appear in separate clades.

**Figure 5:**
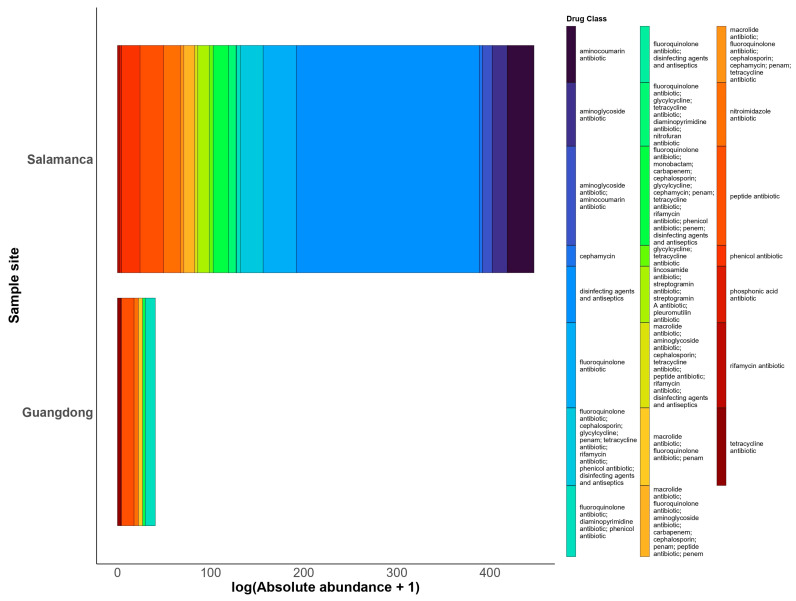
AMR gene classes. Comparison of absolute AMR gene abundance between Salamanca and Guangdong. Bars represent the cumulative abundance of AMR genes expressed as log(RPKm + 1), grouped by antibiotic class. Colors indicate different drug classes. Only genes classes that were detected in contigs, MAGs, and raw reads are included. Genes classes were filtered for quality: contigs with “Strict” or “Perfect” hits, and raw reads with ≥80% coverage.

Antimicrobial resistance gene classes reveals a pattern consistent with that observed at the family level in Figure 5. In Guangdong, the detected AMR gene classes are limited in both diversity and abundance. The most frequently identified include those associated with fluoroquinolones, diaminopyrimidines, phenicols, macrolides, nitroimidazoles, peptides, and phosphonates, exhibiting modest abundances (0.7–13.4 log(RPKM + 1)) and detected in only a small number of samples (2–5).

In contrast, results from Salamanca demonstrate a markedly broader diversity of antibiotic resistance classes and substantially higher abundance levels. Detected classes include genes conferring resistance to aminocoumarins, aminoglycosides, cephamycins, fluoroquinolones, macrolides, tetracyclines, peptides, phenicols, nitroimidazoles, rifamycins, and disinfectants/antiseptics, with abundances ranging from moderate to very high (0.25–196 log(RPKM + 1)). Notably, certain classes, particularly disinfectants/antiseptics, fluoroquinolones, and peptides, are nearly ubiquitous across Salamanca samples.

**Figure 6:**
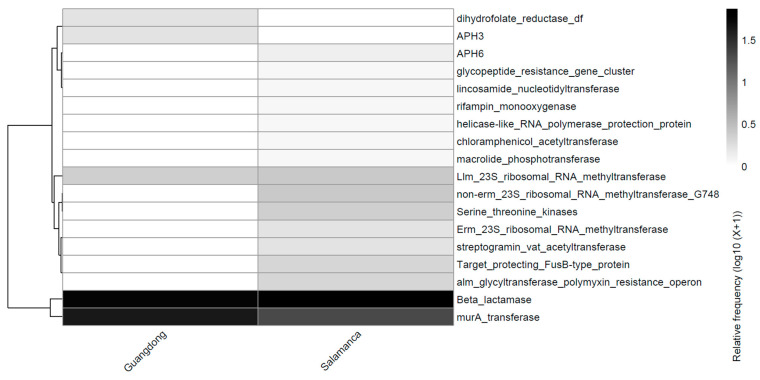
Distribution of antibiotic resistance mechanisms with homologs proteins detected in metagenomic assemblies. The heatmap shows the relative abundance (log10 transformed) of the mechanisms uniquely identified in samples from Guangdong and uniquely detected in Salamanca.

As previously observed in the analysis of genes identified via RGI against the CARD database, the analysis of resistance mechanisms based on detected homologous proteins (Figure 6) further highlights clear distinctions between the Guangdong and Salamanca samples. Both sites share a core set of fundamental resistance mechanisms, including β-lactamases, MurA transferases, and Llm-type 23S ribosomal RNA methyltransferases.

However, samples from Guangdong exhibit only a limited number of additional resistance mechanisms, present at low abundances, such as dihydrofolate reductase (DF) and APH(3’), and completely lack non-Erm-type 23S rRNA modifications. In contrast, samples from Salamanca display a broader repertoire of resistance mechanisms, including Erm-type 23S rRNA methyltransferases, non-Erm 23S rRNA methyltransferase G748, APH(6), among others.

### 2.4. Homologous Mechanisms

**Figure 7:**
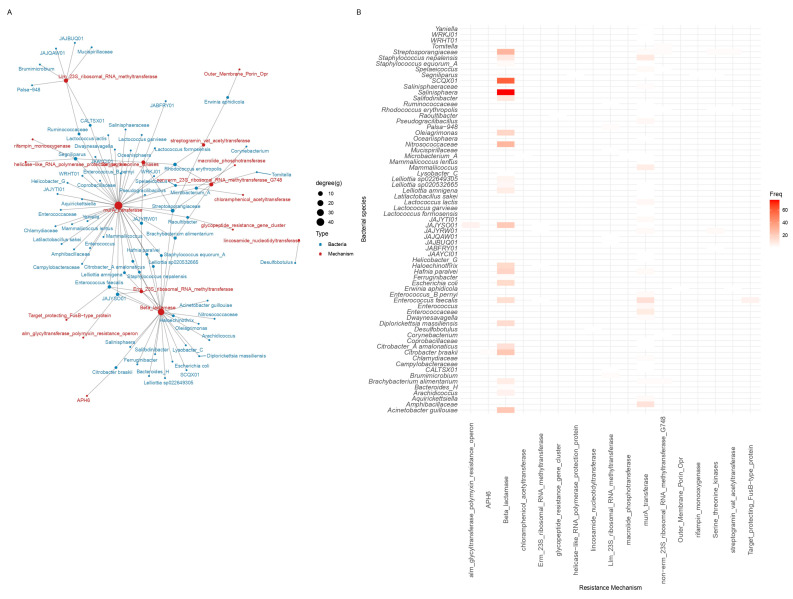
Antibiotic resistance mechanisms for which homologous proteins were uniquely detected in metagenomic assemblies from Salamanca. (**A**) Network of bacterial species and associated ARG mechanisms. (**B**) Frequency of ARG mechanisms across bacterial hosts. Degree (g) indicates the number of connections per node; Freq represents the occurrence frequency.

The analysis of resistance mechanisms based on homologous proteins detected in the metagenomic assemblies (Figure 7) revealed that samples from Salamanca exhibited a richer repertoire of homologous resistance proteins (median richness: 5; Q1: 1, Q3: 13), together with moderate functional diversity (median Shannon index: 1.5; Q1: 0.69, Q3: 2.58). This pattern indicates high overall diversity with low dominance (median Simpson index: 0.8; Q1: 0.5, Q3: 0.92), suggesting that resistance mechanisms in Salamanca are functionally diverse and more evenly distributed across bacterial hosts (Shannon W = 416.5, *p* = 0.0455; Simpson W = 416.5, *p* = 0.0455) [see Supplementary Material File: Homologs_Salamanca and Statistical_results (Alfa_div)].

These include homologous proteins associated with β-lactamases (Abundance: 432; Relative: 71.9%), MurA transferases (Ab: 115; Rel: 19.1%), and Llm-type 23S rRNA methyltransferases (Ab: 9; Rel: 1.5%), followed by a diverse array of additional homologous proteins to resistance mechanisms present at lower frequencies (Ab: 1–8; Rel: 0.16–1.3%). These homologous proteins to resistance mechanisms are distributed across multiple bacterial species, including *Enterococcus faecalis*, *Staphylococcus nepalensis*, *Brachybacterium alimentarium*, *Microbacterium* spp., and *Raoultibacter* spp.

**Figure 8:**
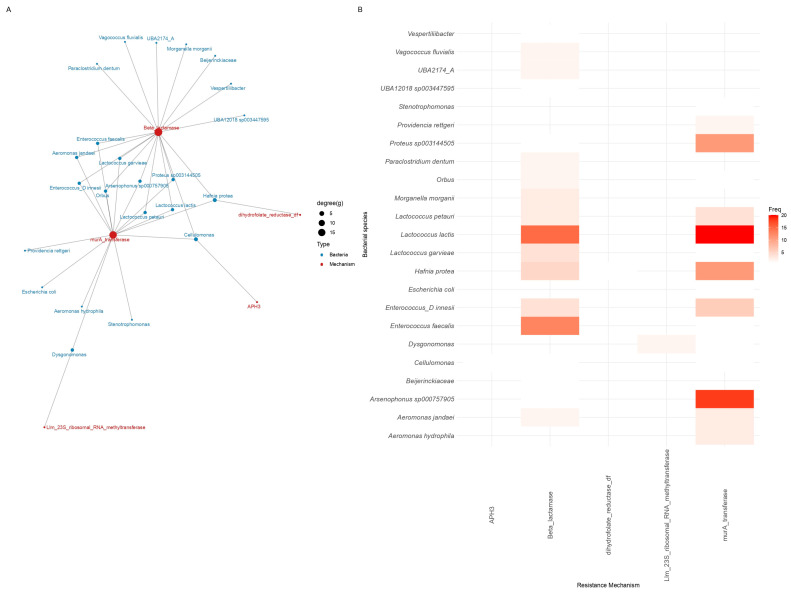
Antibiotic resistance mechanisms for which homologous proteins were uniquely detected in metagenomic assemblies from Guangdong. (**A**) Network of bacterial species and associated ARG mechanisms. (**B**) Frequency of ARG mechanisms across bacterial hosts. Degree (g) indicates the number of connections per node; Freq represents the occurrence frequency.

In contrast, samples from Guangdong (Figure 8) exhibited lower richness (median richness: 3; Q1: 1, Q3: 5) and reduced functional diversity (median Shannon index: 1.1; Q1: 0, Q3: 1.6) compared to Salamanca. The Simpson index (median: 0.66; Q1: 0, Q3: 0.8) also indicated higher dominance, suggesting that only a few resistance mechanisms accounted for most of the detected homologous proteins. This pattern is consistent with a more constrained and uneven distribution of resistance functions within the microbial community of Guangdong. Beta diversity analysis based on Jaccard dissimilarities revealed significant compositional differences in the resistome between the two study sites (PERMANOVA, R^2^ = 0.019, *F* = 1.33, *p* = 0.001). The homogeneity of multivariate dispersions did not differ significantly between groups (*F* = 0.07, *p* = 0.79), indicating that the observed differences are attributable to changes in community composition rather than dispersion heterogeneity. [see Supplementary Material File: Homologs_Guangdong and Statistical_results (Alfa_div, Beta_div)].

Guangdong exhibit a markedly more restricted profile, dominated by homologous proteins to β-lactamases (Ab: 85; Rel: 55.9%) and MurA transferases (Ab: 63; Rel: 41.4%), with other homologous proteins to resistance mechanisms occurring at very low abundances (Ab: 1–2; Rel: 0.65–1.3%) and primarily associated with *Hafnia protea*, *Lactococcus* spp., and *Enterococcus* spp. Notably, several proteins homologous to resistance mechanisms detected in Salamanca, such as fusidic acid target-protecting proteins, Erm and non-Erm 23S rRNA methyltransferases, and modifications conferring resistance to polymyxins or glycopeptides, are entirely absent in the Guangdong dataset.

**Figure 9:**
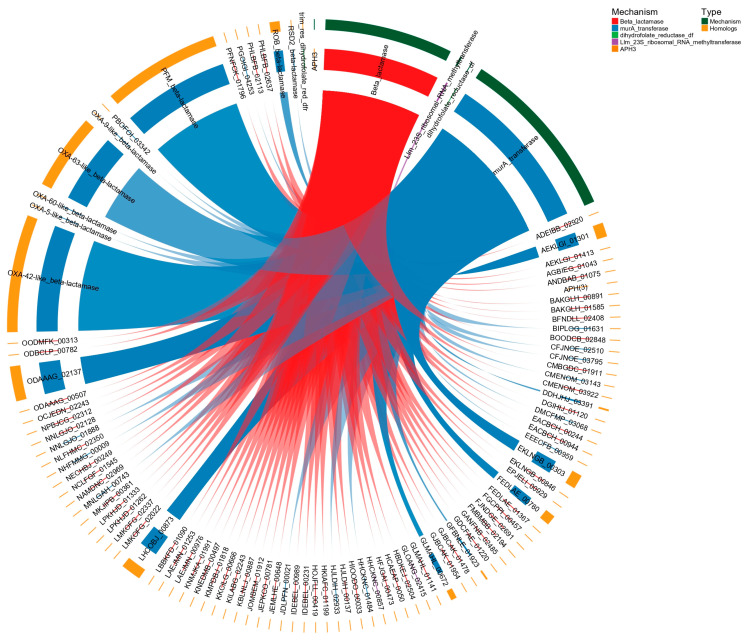
Relationships between CARD resistance mechanisms and their homologs protein “only” detected in Guangdong dataset. Each sector represents either a resistance mechanism (colored according to a predefined palette) or a homolog, and the connecting arcs indicate associations identified via HMMER (full sequence E-value < 0.0001; full sequence score > 200; bias < 0.1).

It is evident that the most representative resistance mechanisms detected in Guangdong dataset (Figure 9) are β-lactamases and MurA transferases, each associated with multiple homologous proteins. Specifically, homologs such as NCLFGF_01545, NNLGJO_02128, and FJNDGE_02691 are linked to β-lactamases, while NNLGJO_01888, AEKLGI_01413, and DDHJHJ_03391 correspond to MurA transferases. Additionally, homologous proteins associated with Llm-type 23S rRNA methyltransferase (GLOANG_02415, HHCKNC_00857), APH(3’) (APH3), and dihydrofolate reductase (trim_res_dihydrofolate_red_dfr) are also detected.

The network of connections reveals that certain homologous proteins are exclusively associated with a specific resistance mechanism, for instance, the multiple links between AEKLGI_01301 and MurA transferase, while other mechanisms exhibit multiple connected homologs which could contribute to functional resistance in these samples.

**Figure 10:**
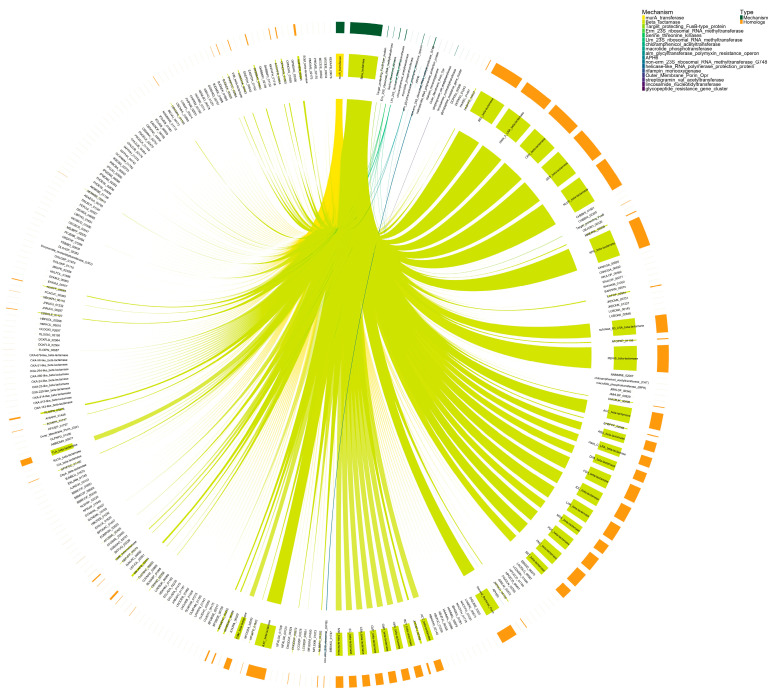
Relationships between CARD resistance mechanisms and their homologs protein “only” detected in Salamanca dataset. Each sector represents either a resistance mechanism (colored according to a predefined palette) or a homolog, and the connecting arcs indicate associations identified via HMMER (full sequence E-value < 0.0001; full sequence score > 200; bias < 0.1).

In Salamanca (Figure 10), as observed in Guangdong, the most abundant resistance mechanisms are also β-lactamases and MurA transferases, although there is a notably greater diversity of subtypes and variants. For instance, multiple homologous proteins are repeatedly associated with β-lactamases, including well-characterized variants such as class_A_LRA, CRH, KPC, KLUC, FOX, PDC, and OXA-like enzymes, reflecting an extensive and functionally redundant repertoire of resistance genes. Indicator species analysis (IndVal.g, 999 permutations) identified two homologous β-lactamase proteins significantly associated with the Salamanca site (IndVal.g = 0.418, *p* = 0.037) [see Supplementary Material File: Statistical_results (Beta_div)]. Similarly, MurA transferases exhibit a high number of detected homologs, including DCPKNJ_00306, HAIKEL_01491, CHBBHI_01901, among many others, which further shows functional diversification within this mechanism.

Moreover, several less abundant but clinically and ecologically relevant resistance mechanisms are also identified, including Erm-type 23S rRNA methyltransferases, Llm-type 23S rRNA methyltransferases, Serine/Threonine kinases, APH(6), and various enzymes associated with polymyxin resistance (alm_glycyltransferase) as well as macrolide and streptogramin acetyltransferases.

Comparison of the results shown in Figure 9 and Figure 10 reveals that, in both the Guangdong and Salamanca datasets, the predominant resistance mechanisms are β-lactamases (*p* < 0.05) and MurA transferases [see Supplementary Material File 17: Statistical_results (Dseq2)]. However, while the Guangdong dataset exhibits a higher frequency of repeated individual homologs (indicating genetic redundancy), the Salamanca dataset displays greater diversity in β-lactamase and MurA transferase subtypes, as well as the presence of additional secondary resistance mechanisms, including 23S rRNA methyltransferases, Ser/Thr kinases, and transferases associated with polymyxin resistance. The bacterial community in Salamanca possesses a more diverse and more adaptable antimicrobial resistance repertoire, capable of responding to a broader range of antibiotic pressures. In contrast, in Guangdong, resistance abundance is concentrated within a more limited set of recurrent homologs, implying a functionally narrower and less flexible resistome.

## 3. Discussion

Our comparative analysis of bacterial communities in Guangdong and Salamanca has provided compelling microbial evidence of anthropogenic pressure gradients. The data, derived from metagenomic sequencing, reveal marked differences in both the abundance and diversity of ARGs and homologous proteins between these locations, suggesting a strong environmental selection pressure driven by human activity. Guangdong’s microbiome remains anchored in natural ecological processes, while Salamanca’s reflects the complex, often risky, blending of environmental and human-associated microbial worlds.

The microbial profile of Guangdong is characterized by a predominance of taxa typically associated with natural aquatic or soil ecosystems, animal commensals, or low-impact environmental reservoirs. Dominant genera such as *Lactococcus* [46], *Aeromonas* [47], *Dysgonomonas* [48], *Stenotrophomonas* [49], and *Paraclostridium* [50] are frequently reported in unpolluted freshwater systems, fish microbiomes, or soil matrices. Species like *Lactococcus lactis* and *L. garvieae* are commonly linked to dairy or aquaculture environments [46], which could mean minimal human fecal or clinical contamination.

Notably, even when potentially pathogenic species such as *Escherichia coli* or *Enterococcus faecalis* appear, their presence is sporadic and likely represents transient environmental colonization rather than sustained anthropogenic input. The absence of hospital-associated or multidrug-resistant taxa (*Acinetobacter*, *Citrobacter braakii*, *Mycobacterium*, *Chlamydiaceae*) further supports the interpretation of Guangdong as an ecosystem with limited human disturbance.

Moreover, the detection of less common or environmentally specialized taxa, such as *Beijerinckiaceae* (methanotrophs) [51], *Vespertiliibacter* (bat-associated) [52] and *Arsenophonus* (insect symbiont) [53], reinforces the hypothesis that Guangdong’s microbiome is shaped primarily by autochthonous ecological processes, rather than human waste or urban runoff.

Although genera such as *Lactococcus*, *Aeromonas*, *Dysgonomonas*, *Stenotrophomonas* and *Paraclostridium* encompass species capable of opportunistic infections in mammals, their low and sporadic detection in the Guangdong samples, together with the dominance of taxa typical of natural aquatic and soil ecosystems, suggests a limited direct impact on bat health under current conditions. Occasional reads assigned to *Escherichia coli* and *Enterococcus faecalis* indicate transient exposure to enteric bacteria, but not a stable colonization by high-risk clinical lineages, which is consistent with a relatively preserved, environmentally driven microbiome and a low immediate public-health concern [54,55].

In contrast, Salamanca’s microbiome presents a dramatically expanded and heterogeneous taxonomic repertoire, integrating environmental, commensal, zoonotic, and clinically human-associated bacteria. The presence of *Enterococcus faecalis* [56], *Escherichia coli* [57], *Citrobacter braakii* [58], *Acinetobacter guillouiae* [59], and *Rhodococcus erythropolis* [60], all recognized opportunistic or nosocomial pathogens, signals direct or indirect contamination from human or livestock sources.

Critically, Salamanca harbors multiple hospital-relevant or emerging pathogen taxa absent in Guangdong, including *Mycobacterium* spp. (potential environmental or clinical strains) [61], *Chlamydiaceae* (obligate intracellular pathogens) [62], *Corynebacterium* [63], *Staphylococcus equorum* [64], *Mammaliicoccus* (skin/mucosal commensals with pathogenic potential) [65], *Lelliottia amnigena* [66], *Hafnia paralvei* (enteric, but increasingly reported in clinical settings) [67].

The co-occurrence of environmental genera (*Salinisphaera*, *Psychrobacter*) [68,69] with human-associated taxa suggests ecological mixing zones, likely driven by wastewater discharge, agricultural runoff, or urban effluents. This blending creates ideal conditions for horizontal gene transfer (HGT), particularly of ARGs, between environmental reservoirs and clinically relevant strains.

Furthermore, the presence of fecal indicators (*Enterococcaceae*, *Bacteroides_H*, *Coprobacillaceae*) and gut-associated commensals (*Ruminococcaceae*, *Lachnospiraceae*, *Campylobacteraceae*) strongly implies contamination from human or animal gastrointestinal tracts, a hallmark of anthropogenic impact [70].

In Salamanca, the higher prevalence of *Enterococcus faecalis*, *Escherichia coli*, *Citrobacter braakii*, *Acinetobacter guillouiae* and *Rhodococcus erythropolis* is more worrying from an animal-health perspective, because these genera include well-known agents of septicemia, urinary and gastrointestinal infections in mammals, and several studies have documented multidrug-resistant enterobacteria and staphylococci in bats. Asymptomatic bats carrying high loads of potentially pathogenic enteric bacteria are increasingly recognized as “pathogen bloomers” and mobile reservoirs that can disseminate diarrheagenic and invasive strains across large spatial scales, particularly in urban or agricultural landscapes, thus creating plausible spillover routes to humans and livestock [55,71].

We found an elevated abundance of the *qacG* gene across nearly all Salamanca samples. This gene, which encodes a small multidrug resistance (SMR) efflux pump conferring resistance to quaternary ammonium compounds (QACs), is frequently associated with disinfectant and biocide exposure [72], substances ubiquitously employed in clinical, agricultural, and domestic settings. In Salamanca, *qacG* consistently exhibits high RPKm_log values (ranging from 3.35 to 10.10), often dominating the resistome profile of individual samples. Notably, in several samples (SRR33769960, SRR33769961), *qacG* reaches its maximum observed expression, suggesting strong and chronical selective pressure from disinfectants (QACs) or related compounds in the environment.

In our assembly, qacG-positive contigs were taxonomically assigned to both environmental and opportunistic taxa, including *Staphylococcus* (predominantly *S. nepalensis*), *Enterococcus faecalis*, *Escherichia coli* and enteric *Gammaproteobacteria* such as *Citrobacter braakii*, *Lelliottia* spp. and *Hafnia paralvei*, as well as several additional genera that could only be resolved at genus level. This taxonomic breadth indicates that qacG is not restricted to a single clinical lineage but circulates among diverse bacterial groups inhabiting bat guano. From an animal-health perspective, the presence of qacG in gut and environment-associated bacteria colonizing bats may prolonged carriage of opportunistic pathogens in the intestine and in roost biofilms, potentially contributing to chronic or recurrent subclinical infections and increased bacterial loads in guano [73,74,75]. In terms of human health, bats commuting over tunnels with bacteria that present this gen may act as mobile reservoirs of qacG-positive *Staphylococcus*, *Enterococci* and *Enterobacteria* that already show tolerance to widely used disinfectants; such strains, if transmitted to humans or livestock, could undermine disinfection-based infection-control strategies and co-select for antibiotic resistance [76].

In contrast, *qacG* is entirely absent from the Guangdong dataset. This absence strongly supports the hypothesis that anthropogenic inputs, particularly the use of disinfectants and antiseptics, are driving the selection and proliferation of *qacG*-harboring microorganisms in Salamanca. The lack of such pressure in Guangdong likely precludes the maintenance of this energetically costly resistance determinant in the absence of consistent selective advantage.

Beyond *qacG*, Salamanca exhibits a more complex and diverse resistome. Multiple additional ARGs are recurrently detected. Beyond biocide resistance, Salamanca exhibited enrichment of ARGs conferring resistance to critically important antibiotic classes for human medicine. Curiously, all the samples with ARGs detected were from bacterial communities inhabiting aged, desiccated bat guano that accumulates on cave floors, forming stratified deposits [45]. Notably genes associated with fluoroquinolone resistance, including members of the major facilitator superfamily (MFS) and resistance-nodulation-cell division (RND) efflux systems, were recurrently detected and often co-occurred with global transcriptional regulators (*marA*, *ramA*), suggesting the activation of coordinated, multidrug resistance phenotypes [77,78,79]. Additionally, the presence of *tet(X)*, an enzymatic inactivator conferring resistance to last-resort glycylcycline antibiotics such as tigecycline, at moderate abundance (RPKm_log = 4.20; SRR33769962) raises public health concerns, as this gene is both mobile and capable of undermining last-line therapeutic options [80,81].

In a bat microbiome context, the co-occurrence of MFS/RND efflux determinants with marA/ramA suggests that bats foraging in anthropogenically impacted habitats may carry bacterial populations that are already pre-adapted to withstand clinically used fluoroquinolones and multiple unrelated antimicrobials, which increases the likelihood that these animals act as environmental amplifiers and long-distance dispersers of multidrug-resistant strains [27].

Resistance to phenicol antibiotics was also prominent in Salamanca, mediated by chloramphenicol acetyltransferases (CATs) with RPKm_log values reaching 6.36 (SRR33769968). Given the restricted clinical use but continued veterinary application of chloramphenicol, this signal likely reflects agricultural or wastewater contamination [82]. Similarly, high abundance of *lsaD* (RPKm_log = 6.42; SRR33769984), an ABC-F transporter conferring cross-resistance to lincosamides, streptogramins, and pleuromutilins, further implicates livestock-associated antibiotic use as a key anthropogenic driver [83,84].

For bats, carriage of ABC-F-positive commensals or opportunistic pathogens may prolong intestinal colonization and increase the risk of subclinical infections that are refractory to lincosamide- or pleuromutilin-based treatments, should these drugs ever be needed in wildlife or rehabilitation settings. From a human and public-health perspective, ABC-F-mediated resistance to LSA-P antibiotics in wildlife-associated bacteria is worrisome because these agents are important alternatives against multidrug-resistant Gram-positive infections, so their erosion in environmental reservoirs carried by bats could further narrow therapeutic options and complicate efforts to contain antimicrobial resistance within a One Health framework [27,85,86].

In contrast, the resistome of Guangdong was markedly less complex and dominated by genes associated with intrinsic or environmentally adaptive resistance. Most notably, *ArnT*, a phosphoethanolamine transferase conferring resistance to cationic antimicrobial peptides via lipid A modification [87], was the most abundant ARG in Guangdong (RPKm_log = 6.81; SRR24146884), likely reflecting adaptation to natural stressors such as metal ion limitation or microbial competition rather than exposure to clinical antibiotics. The sporadic detection of *tet(36)* (ribosomal protection; RPKm_log = 3.73) and low levels of *FosA8* further supports the interpretation that resistance in Guangdong arises from ecological pressures rather than anthropogenic selection [88,89].

The detection of *CMY-101*, a plasmid-borne β-lactamase conferring resistance to extended-spectrum cephalosporins, exclusively in Salamanca (RPKm_log = 3.45; SRR33769978) underscores the role of human-impacted environments as reservoirs for mobile, clinically relevant resistance determinants [22]. Collectively, these findings demonstrate that anthropogenic activity not only amplifies the abundance of ARGs but also shifts the resistome toward mechanisms of direct clinical concern, including those conferring resistance to WHO “Highest Priority Critically Important Antimicrobials [90].

In both the Salamanca (Spain) and Guangdong (China) metagenomic datasets, β-lactamase and MurA transferase homologous proteins were identified as the most abundant antimicrobial resistance (AMR) mechanisms, indicating their foundational role in the resistance profiles of the respective bacterial communities. This shared predominance suggests that resistance to β-lactam antibiotics and cell wall biosynthesis inhibitors is a conserved adaptive trait across both environments [91,92]. Previous studies in bats have identified both β-lactam resistance mechanisms (ESBL enzymes) and aminoglycoside-related mechanisms. For example, aminoglycoside resistance genes were observed in addition to β-lactam resistance genes in *Nyctalus noctula* and *Vespertilio murinus* [93]. In a systematic analysis, genes such as aph(3′)-iIa (kanamycin) and aac(3)-II (gentamicin) were detected in wild bats [94]. In contrast, studies of fruit bats in Australia showed a clear dominance of β-lactam resistance in colonies exposed to urban environments [94].

In Salamanca, β-lactamase homologous proteins are represented by a wide array of molecular subtypes (including clinically variants such as KPC, KLUC, OXA-like, FOX, PDC, and class_A_LRA) demonstrating a highly diversified and functionally redundant gene pool. Similarly, MurA transferases in this dataset are associated with numerous distinct homologous proteins (DCPKNJ_00306, HAIKEL_01491, CHBBHI_01901), further underscoring functional diversification within this pathway. This heterogeneity suggests not only exposure to a broad spectrum of β-lactam antibiotics but also sustained selective pressure driving the maintenance and diversification of resistance determinants. In contrast, the Guangdong dataset, while exhibiting comparable overall abundance of β-lactamases and MurA transferases, displays markedly lower subtype diversity. Resistance in this location is characterized by repeated detection of identical or near-identical homologs (such as AEKLGI_01301, which is recurrently linked to MurA transferase) indicating high gene redundancy rather than functional innovation. This pattern may reflect environmental homogeneity, or selection for a limited set of highly efficient resistance determinants.

Beyond these core mechanisms, Salamanca exhibits a broader repertoire of secondary resistance determinants. The presence of these diverse, often clinically relevant mechanisms indicates a complex, multi-layered resistome capable of countering a wide range of antimicrobial agents. In contrast, Guangdong shows minimal representation of such secondary mechanisms, suggesting a functionally narrower resistance landscape.

The monophyletic clustering of Salamanca sequences with reference ARGs suggests functional conservation of resistance mechanisms, rather than novel or divergent adaptations. This implies that selective pressures in the anthropized environment maintain canonical resistance determinants already circulating in clinical reservoirs. The close phylogenetic relationship with reference ARGs may indicate recent horizontal gene transfer from anthropogenic sources, consistent with the observed enrichment of clinically relevant resistance genes in the Salamanca samples. While monophyletic clustering implies shared ancestry and putative orthology, further functional validation (protein modeling or expression assays) is needed to confirm conserved resistance phenotypes.

We acknowledge that this study is based on only two sampling sites, which limits our ability to fully disentangle the effects of anthropogenic exposure from broader physiogeographic variation. In this context, PERMANOVA analysis revealed statistically significant differences in resistome composition between sites, but with a low proportion of variance explained by location (R^2^ = 0.019). Such low effect sizes are common in high-dimensional resistome datasets and indicate that anthropogenic exposure likely represents only one of several interacting factors shaping resistome structure.

Differences between sites in climate, landscape structure, regional microbial pools, and bat species composition may independently influence microbiome and resistome profiles; therefore, caution is warranted when extrapolating our findings beyond the studied systems.

Nevertheless, both locations are characterized by cave-dwelling, insectivorous bat assemblages dominated by genera such as *Miniopterus*, *Myotis*, and *Rhinolophus*, which exhibit comparable roosting behaviors and trophic niches. This ecological convergence provides a partial framework for interregional comparison, allowing us to examine how differing levels of human disturbance may shape bat-associated resistomes under broadly similar life-history strategies.

Notably, the Spanish colony represents a system subjected to multifactorial anthropogenic pressures, including tourism, guano harvesting, and chemical disinfection, whereas the Chinese colony constitutes a near-pristine reference condition. Within this context, our results should not be interpreted as definitive, but rather as suggestive evidence that sustained human presence may amplify the diversity, homogeneity, and clinical relevance of resistance genes within wildlife-associated microbiomes. Future studies incorporating multiple sites per disturbance category, broader-scale environmental metadata, and host-specific controls will be essential to disentangle anthropogenic effects from underlying biogeographic variation and robustly assess the generality of these patterns.

Our findings emphasize the need for structured environmental AMR surveillance programs in wildlife habitats under human influence. Such monitoring should include periodic sampling of bat guano (biannually, during breeding and non-breeding seasons) to detect temporal fluctuations in ARG abundance and diversity. Key indicators should comprise the relative abundance of clinically relevant genes such as bla, aac, and tet families, the presence of mobile genetic elements (integrons, plasmids), and quantification of human-associated bacterial taxa. Environmental metadata, including disinfectant use, visitor density, and microclimatic parameters, should be recorded in parallel to contextualize changes in the resistome.

To mitigate anthropogenic impacts, management strategies should prioritize minimizing direct human–bat contact and reducing chemical disturbance. Alternatives to broad-spectrum disinfectants (physical cleaning, enzymatic or low-toxicity biocides) could be evaluated to limit co-selection of resistance genes. Controlled access to bat roosting areas, seasonal closures during breeding periods, and awareness programs for park personnel and tourists would further help to balance conservation, tourism, and public health goals.

## 4. Materials and Methods

The Camino del Hierro is situated within the Arribes del Duero Natural Park. The region features terraced hills once used for traditional agriculture, supported by dry-stone walls. Many of these terraces are now abandoned, giving way to a mosaic of mixed woodlands, scrub, and grasslands. [95]. Terraces that remain in cultivation typically host olive and almond groves, although natural reforestation with oak, holm oak, and juniper is widespread due to ongoing land abandonment. The broader landscape includes valleys between mountain ranges, elevated plateaus, and dramatic granite cliffs carved by a Duero River tributary, with vertical drops reaching up to 500 m [95]. Elevation across the area ranges from 100 to 2000 m above sea level. The climate is classified as Mediterranean-continental with semi-arid characteristics, receiving less than 400 mm of rainfall annually. Temperatures can range from −10 °C in winter to 40 °C in summer, although the interior of the Camino del Hierro’s tunnels maintains relatively stable thermal conditions [96]. In the 19th century, a railway line known as “Camino de Hierro”, and formed by 20 tunnels and 10 bridges, was constructed within the park. After being abandoned for over 40 years, two tunnels of Camino de Hierro have become an important refuge for several bat species. Despite the presence of clinically relevant bacteria in bat guano, both bat-inhabited tunnels of “Camino del Hierro” were opened as an ecotourism attraction in 2021 receiving over 50,000 visitors since then [43].

On the other hand, samples from Guangdong were collected in Yanyan Cave, Huizhou City, Guangdong Province, China (23.19018° N, 114.81386° E) [97]. The study area is located in southern China, in an inland part of Guangdong. The landscape consists of low mountains and hilly terrain interspersed with river valleys, traditionally used for small-scale agriculture such as rice paddies, fruit orchards, and terraced fields [98]. Many of these areas are gradually being abandoned, allowing secondary forest and scrub to expand over former croplands. Natural vegetation includes subtropical broadleaf forest with species of oak, camphor, and bamboo, although human disturbance has resulted in a mosaic of woodland, shrubland, and agricultural patches [98]. The subtropical monsoon climate is characterized by warm, humid summers with abundant rainfall and mild, relatively dry winters [98,99]. Elevation ranges from approximately 50 to 1200 m above sea level, with steep slopes shaping local hydrology [99]. Unlike recognized natural parks or ecotourism destinations, this area does not receive organized visits from tourists, and land use is primarily local and subsistence-oriented [100].

### 4.1. Sample Collection

Fecal material was collected from bat colonies inhabiting two tunnels along the Camino del Hierro: tunnel 1 (“La Carretera”, 1500 m) and tunnel 3 (“Morgado”, 423 m) described in Soto-López [45]. In brief, a total of 48 fresh droppings were collected from 1 m^2^ sampling grids placed beneath roosting sites, using sterile scoops to collect material from the top 5–10 cm at five randomly selected points per grid [see Supplementary Material File: Sample_inf]. Samples were placed in sterile 50 mL Falcon tubes for transport and storage at −20 °C.

Species identification of the bat colonies was initially based on photographic assessment of morphological traits as described by García-Martín [44], and later validated through molecular and bioinformatic methods. Molecular confirmation is described in García-Martín et al. (2025) [44]. Briefly, targeting a ~202 bp region of the mitochondrial COI gene using primers SFF-145f and SFF-351r. PCR conditions included an initial denaturation at 95 °C (2 min), 35 cycles at 95 °C (1 min), 65 °C (1 min), and 72 °C (2 min), with a final extension at 72 °C for 10 min. Resulting amplicons were sequenced (Sanger), and species were assigned via BLAST comparison against the NCBI database.

In addition, host genetic material was filtered by aligning sequences against reference genomes for *Myotis myotis*, *Rhinolophus ferrumequinum*, and *Miniopterus schreibersii*, downloaded from NCBI using the ncbi-datasets-cli v14.0.0 (https://www.ncbi.nlm.nih.gov/datasets/docs/v2/command-line-tools/download-and-install/) [101], and aligned using Bowtie2 v2.5.4 (https://www.metagenomics.wiki/tools/short-read/remove-host-sequences) [102,103].

Sample collection was carried out in accordance with the Bioethics Committee of the Universidad de Salamanca (ref. RD 53/013, registration no. 965/2023) and with the authorization of the Servicio Territorial de Medio Ambiente (Delegación Territorial de Salamanca, Junta Castilla y León, Spain), under license AUES_SA_12 (JC)_23. All experiments were conducted in accordance with biosafety level 2 laboratory guidelines.

### 4.2. DNA Extraction and Sequencing

As described in Soto-López [45], genomic DNA was extracted directly from guano samples using the DNeasy^®^ PowerSoil^®^ Pro Kit (Qiagen, Barcelona, Spain), with slight modifications including a final elution volume of 50 µL and mechanical lysis via TissueLyser II. DNA concentration was assessed using a Nanodrop ND-1000 spectrophotometer (Nanodrop Technologies, USA) and visualized on 1.5% agarose gels under a UVP Biodoc-It^®^ 2 system (Analytik Jena, Germany). Extracted DNA was stored at −20 °C until further processing.

For sequencing, 48 libraries were prepared from 200 ng of DNA each, employing the xGen™ DNA Library Prep EZ Kit (Integrated DNA Technologies, Belgium). Library quality was evaluated using the Invitrogen™ Qubit™ 3 Fluorometer and the Agilent 2100 Bioanalyzer (Agilent Technologies, Germany). Libraries were sequenced via paired-end 150 bp reads with dual indexing on the Illumina^®^ NovaSeq X Plus platform (Illumina, USA) at the DNA sequencing service of the University of Salamanca (NUCLEUS, platform, University of Salamanca). Negative controls (sterile ddH_2_O) were included throughout, yielding no detectable reads. All procedures were performed under strict contamination control using DNase-free consumables to ensure sample integrity.

### 4.3. Study Selection and Data Retrieval of Control Data

To identify relevant metagenomic projects for downstream analysis, we queried the Sequence Read Archive (SRA) database at the National Center for Biotechnology Information (NCBI). A comprehensive search strategy was designed using a combination of keyword-based queries combined with filters based on Boolean logic to maximize the retrieval of bat-associated metagenomic studies.

The Boolean search terms included: “bat metagenome”[Organism] AND (cluster_public[prop] AND “biomol dna”[Properties] AND “strategy wgs”[Properties] AND “filetype fastq”[Properties]) AND (cluster_public[prop] AND “biomol dna”[Properties] AND “strategy wgs”[Properties] AND “library layout paired”[Properties] AND “platform illumina”[Properties]).

Additionally, we used the query: “bat”[All Fields] AND “metagenome”[All Fields] AND “WGS”[All Fields].

Search results were filtered by organism (*Chiroptera*), library layout (paired-end), biomolecule type (DNA), sequencing strategy (whole-genome shotgun), and file format (FASTQ). No date restrictions were applied. A total of 878 records were initially retrieved.

Subsequently, a manual curation step was performed to refine the dataset. Only projects involving biological samples from bats, specifically fecal material or intestinal content, were selected. Studies focusing on targeted amplification of specific genomic regions were excluded in favor of those employing whole-genome shotgun (WGS) approaches. Additionally, projects lacking geographic sampling information or metadata regarding sample origin were discarded. Furthermore, all studies primarily targeting viromes or employing viral enrichment strategies were excluded using the SRA Run Selector tool.

With this approach we selected the project with the identification number PRJNA954561, “Metagenome of bat: *Rhinolophus macrotis*” and used the samples described by Guo et al. (2023) [97].

### 4.4. Metagenome Preprocessing and Assembly

With both projects’ data, we trimmed Low-quality sequences and primers using Trim Galore v0.6.10 (--phred33 --length 100 --stringency 3 --paired --fastqc) (https://www.bioinformatics.babraham.ac.uk/projects/trim_galore/) [104]. We also deleted multi-G and multi-A sequences adding the sequence of interest to the command (--adapter GGG…). Then we aligned and removed the potential host sequences using Bowtie2 v2.5.4 (https://www.metagenomics.wiki/tools/short-read/remove-host-sequences) [102,103]. Duplicated reads were deleted using Fastp v1.0.1 (--length_required 100 --qualified_quality_phred 20 –dedup) (https://github.com/OpenGene/fastp) [105]. Quality control was done with fastqc (https://www.bioinformatics.babraham.ac.uk/projects/fastqc/) [106].

Deduplicated reads were assembled into contigs using MEGAHIT v1.2.9 (https://github.com/voutcn/megahit) [107]. Contigs longer than 1500 bp were filtered using SeqKit v2.10.0 (-m 1500) (https://github.com/shenwei356/seqkit) [108] and subsequently aligned to the raw reads using Bowtie2. Reads that did not map to the Bowtie2-generated index were digitally normalized using BBNorm de BBmap v39.26 (target = 40 min = 5 passes = 2) (https://github.com/BioInfoTools/BBMap) [109]. For samples 90, 91, and 92 from our project (PRJNA1269778), an additional preprocessing step was required prior to digital normalization, consisting of quality correction using Fastp (--detect_adapter_for_pe --trim_front1 10 --trim_front2 10).

The normalized raw reads were assembled using metaSPAdes de Spades v4.2.0 (-m 44 -k 21,33) (https://github.com/ablab/spades) [110]. Contigs longer than 1500 bp were filtered with SeqKit and merged with those previously obtained from MEGAHIT. This new set of contigs (>1500 bp) was again mapped to the raw reads using Bowtie2. Mapped reads were sorted and indexed using Samtools v1.22 (https://github.com/samtools/samtools) [111], and coverage depth was calculated with jgi_summarize_bam_contig_depths from MetaBAT 2 v2.18 (https://bitbucket.org/berkeleylab/metabat/src/master) [112]. These coverage profiles were then used to bin the contigs with MetaBAT 2. The same sorted and indexed raw reads were also used to estimate abundance with jgi_summarize_bam_contig_depths and to perform binning with MaxBin v2.2.7 (https://sourceforge.net/projects/maxbin) [113].

In parallel, contigs >1500 bp from the combined MEGAHIT and metaSPAdes assemblies were fragmented into segments of up to 10,000 bp using cut_up_fasta.py from CONCOCT v1.1.0 (https://github.com/BinPro/CONCOCT) [114]. Coverage of these fragmented contigs was then calculated by mapping the same raw reads (sorted and indexed) using concoct_coverage_table.py, generating a coverage table. Fragmented contigs and the coverage table were then used for binning with CONCOCT.

The scaffolds obtained from the three binning tools (MetaBAT2, MaxBin, and CONCOCT) were dereplicated and refined using DAS Tool v1.1.7 (--search_engine diamond --write_bins) (https://github.com/cmks/DAS_Tool) [115] to extract consensus bins. The resulting bins were evaluated for completeness and contamination using CheckM2 v1.1.0 (https://github.com/chklovski/CheckM2) [116] (database: uniref100.KO.1.dmnd) to assess the quality of prokaryotic bins, and with BUSCO v6.0.0 (https://busco.ezlab.org/busco_userguide.html) [117] (database: fungi_odb12) to evaluate potential fungal eukaryotic bins. Bins passing the CheckM2 quality filter (min_comp = 90, max_cont = 5) were retained. No fungal bins were identified in the BUSCO evaluation. The filtered bins were annotated using BAKTA v1.11.3 (--type full) (https://github.com/oschwengers/bakta) [118].

The CARD (Comprehensive Antibiotic Resistance Database) v4.0.1 (https://card.mcmaster.ca/download) [30] was loaded, and genes associated with antimicrobial resistance were grouped by resistance mechanism, based on column 9 of the aro_index.tsv file [see Supplementary Material File: Reference_data]. These grouped resistance mechanisms were aligned using MAFFT v7.526 (https://mafft.cbrc.jp/alignment/software/linux.html) [119], and the resulting alignments were used to construct HMM profiles (.hmm files) with hmmbuild from HMMER v3.4 (http://hmmer.org) [120].

Next, the bacterial species used in the construction of the CARD database (listed in column 1 of aro_index.tsv) were identified [see Supplementary Material File: Reference_data], and their complete genomes (reference genomes) were downloaded in compressed format using the NCBI Datasets CLI v18.5.0 (https://github.com/ncbi/datasets) [101]. These bacterial genomes were also annotated using BAKTA.

Homologous resistance genes were searched within both the assembled bins and the reference bacterial genomes from CARD. Searches were performed using hmmsearch (HMMER) for mechanisms containing multiple genes, or phmmer (HMMER) when only a single gene was associated with a mechanism [see Supplementary Material File: Reference_data]. Protein domains of the homologous sequences identified by HMMER. Homologous proteins to CARD genes were concatenated with their counterparts from the reference genomes and aligned using MAFFT. The resulting alignments were refined with trimAL v1.5.0 (-automated1) (https://github.com/inab/trimal) [121], and phylogenetic trees for each CARD resistance mechanism were inferred using IQ-TREE v3.0.1 (https://iqtree.github.io) [122]. Maximum likelihood phylogenetic trees were constructed for Aminoglycosides (concatenated sequences -cs-: 77, amino acid sites -aams-: 220, informative -is-: 219), (cs: 4387, aams: 204, is: 204), Colistin/phosphoethanolamine transferases (cs: 752, aams: 312, is: 312), Fosfomycin transferases (cs: 213, aams: 416, is: 411), Glycopeptides (cs: 7783, aams: 97, is: 97), Macrolide phosphotransferase (cs: 23, aams: 282, is: 271), methicillin (cs: 40, aams: 522, is: 481), Ribosomal RNA methyltransferases (cs: 350, aams: 335, is: 335), Rifampicins (cs: 62, aams: 248, is: 245), Sulfonamides_DHPS (cs: 248, aams: 268, is: 251), Tetracyclines (cs: 2546, aams: 326, is: 325) in Salamanca data. We used evolutionary model LG+F+G4 for all the protein trees. Node support was estimated with 1000 ultra-fast bootstrap replicates [123] and 1000 approximate likelihood-ratio tests. For the outer groups used [see Supplementary Material File: Reference_data].

Bins filtered with CheckM2 were annotated with Prokka v1.14.6 (https://github.com/tseemann/prokka) [124], taxonomically classified using GTDB-Tk v2.3.2 (https://github.com/Ecogenomics/GTDBTk) [125] in KBase v1.0.1 (https://www.kbase.us) [126], and a species-level phylogenetic tree was constructed alongside the CARD reference genomes using BUSCO proteins conserved (bacteria_odb12) aligned with MAFFT, refined with trimAL, in IQ-TREE. Maximum likelihood phylogenetic tree was constructed using an alignment of 236 concatenated sequences with 34,780 amino acid sites (31,448 informative). The best evolutionary model identified by ModelFinder was LG+F+G4. Node support was estimated with 1000 ultra-fast bootstrap replicates and 1000 approximate likelihood-ratio tests [see Supplementary Material File: sp_phylo_tree_ufboot]. For the outer groups used [see Supplementary Material File: Reference_data].

We searched for antimicrobial resistance genes (ARGs) in bins, MAGs, and raw reads from the Salamanca and Guangdong samples using the Resistance Gene Identifier (RGI) v6.0.5 [30] against the CARD database. To ensure data quality, we filtered the results as follows: for contigs, only hits classified as “Strict” or “Perfect” were retained; for raw reads, only hits with ≥80% coverage were included. Abundances were then normalized to RPKm (reads per kilobase per thousand mapped reads; note that we used 1000 rather than 1,000,000 as the scaling factor) and subsequently transformed using log(RPKm + 1). Finally, the processed data were visualized using ggplot2 v 4 [127] y ggtree v 3.16.3 [128] in R v 4.5.1 [129].

### 4.5. Methodology for Diversity and Differential Abundance Analysis

Alpha and beta diversity analyses, along with differential abundance testing of homologous antimicrobial resistance mechanisms, were performed in the R, using the following packages: dplyr v 1.1.4 [130], tidyr v 1.3.1 [131], vegan v 2.7-1 [132], indicspecies v 1.8.0 [133], DESeq2 v 1.48.2 [134], and apeglm v 1.30.0 [135]. Homology tables derived from metagenomic assemblies from Guangdong and Salamanca were merged and labeled according to their geographic origin. A binary presence–absence matrix of homologous proteins per sample was generated using the table() function. Presence values (>0) were converted to 1 to indicate the occurrence of each homolog in each sample.

Species richness (the number of unique homologs) and Shannon and Simpson diversity indices were computed using the diversity() function from the vegan package applied to the transposed presence–absence matrix. Differences in diversity indices between groups (Guangdong vs. Salamanca) were assessed using Wilcoxon rank-sum tests.

Dissimilarities in resistance mechanism composition among samples were quantified using the Jaccard distance, computed via the vegdist() function (method = “jaccard”). A permutational multivariate analysis of variance (PERMANOVA) was conducted using the adonis2() function (999 permutations) to test for significant differences between geographic groups. Multivariate dispersion homogeneity (homogeneity of multivariate variances) was verified using the betadisper() function followed by an ANOVA.

The indicspecies package was employed to identify resistance mechanisms significantly associated with each geographic group based on their frequency and specificity. The multipatt() function (999 permutations) was used for this purpose.

Homolog counts were aggregated by resistance mechanism family using dplyr functions and pivot_wider(). The resulting count table was combined with sample metadata to construct a DESeqDataSet object (DESeqDataSetFromMatrix()), with the design formula ~ Group. Given the sparsity of the data, size factors were estimated using the “poscounts” method. A negative binomial generalized linear model was fitted using the DESeq() function with fitType = “parametric”. Differential abundance results for the contrast Salamanca vs. Guangdong were extracted using the results() function, and log_2_ fold changes were subsequently refined using apeglm shrinkage (lfcShrink(type = “apeglm”)).

## 5. Conclusions

Taken together, these findings point to two contrasting ecological and evolutionary scenarios in bat-associated resistomes, with direct implications for animal and public health. The Salamanca microbial community appears to harbor a more adaptable, resilient, and genetically diverse resistome, potentially shaped by heterogeneous and sustained antimicrobial pressures. These may translate into a greater capacity of bat-associated bacteria to withstand therapeutic interventions and to act as reservoirs and dispersal agents of multidrug-resistant pathogens at the wildlife–human interface. In contrast, the Guangdong community, while enriched in core resistance mechanisms, exhibits lower functional and genetic diversity, which limit the risk of dissemination of complex resistance profiles.

These interpretations must be considered within the context of unavoidable physiogeographical differences between the two study sites, including climate, landscape, regional microbial pools, and host community composition, all of which may independently influence microbiome and resistome structure. Overall, our results meet the initial objective of characterizing bat-associated resistomes along an anthropogenic pressure gradient and highlight bats in highly impacted landscapes such as Salamanca as relevant sentinels and potential amplifiers of antimicrobial resistance of concern for both animal health and human public health under a One Health framework.

## Data Availability

The datasets supporting the conclusions of this article are included within the article and its supplementary files. Raw sequencing data are available in the Sequence Read Archive (SRA) under BioProject accession number PRJNA1269778, with BioSample accession numbers: SAMN48801055, SAMN48801056, SAMN48801057, SAMN48801058, SAMN48801059, SAMN48801060, SAMN48801061, SAMN48801062, SAMN48801063, SAMN48801064, SAMN48801065, SAMN48801066, SAMN48801067, SAMN48801068, SAMN48801069, SAMN48801070, SAMN48801071, SAMN48801072, SAMN48801073, SAMN48801074, SAMN48801075, SAMN48801076, SAMN48801077, SAMN48801078, SAMN48801079, SAMN48801080, SAMN48801081, SAMN48801082, SAMN48801083, SAMN48801084, SAMN48801085, SAMN48801086, SAMN48801087, SAMN48801088, SAMN48801089, SAMN48801090, SAMN48801091, AMN48801092, SAMN48801093, SAMN48801094, SAMN48801095, SAMN48801096, SAMN48801097, SAMN48801098, SAMN48801099, SAMN48801100, SAMN48801101, SAMN48801102. The analysis pipeline used in this study is available at: https://github.com/jdjuliosoto/resistome_eIntro2025. The Metagenome-Assembled Genomes are available at: https://doi.org/10.5281/zenodo.17280657.

## Supplementary Materials

The following supporting information can be downloaded at: https://www.mdpi.com/article/10.3390/antibiotics15010051/s1, Table S1: Sample_inf; Table S2: Mags_report; Table S3: Homologs_Guangdong; Table S4: Homologs_Sala; Table S5: Reference_data; Table S6: Statistical_results; Figure S1: Aminoglycosides; Figure S2: β-lactamases; Figure S3: Colistin_phosphoethanolamine_transferases; Figure S4: Fosfomycin_transferases; Figure S5: Glicopeptides; Figure S6: Macrolide_phosphotransferase; Figure S7: Methicillin; Figure S8: Ribosomal_RNA_methyltransferases; Figure S9: Rifampicin; Figure S10: Sulfonamides_DHPS; Figure S11: Tetracyclines.
