# Supplementary material for: Metagenomic Comparison of Bat Colony Resistomes Across Anthropogenic and Pristine Habitats
Source: Antibiotics (Basel). 2026 Jan 3;15(1):51. doi: 10.3390/antibiotics15010051 (PMC12838372; doi:10.3390/antibiotics15010051)
Supplement: Supplementary file 1 [file antibiotics-15-00051-s001.zip › Supplementary file captions.pdf]

Supplementary file captions

Additional File 1. Sample information.

Sequencing information including sample id, BioProject, localization number of reads pre and post preprocessing and sequence length.

Additional File 2. MAGs report.

Taxonomical and quality report of the metagenome-assembled genomes from Salamanca and Guangdong.

Additional File 3. Homologous Guangdong.

Homologous proteins inferred by Hmmer, with full sequence E-value < 0.0001, full sequence score > 200 and bias < 0.1.

Additional File 4. Homologous Salamanca.

Homologous proteins inferred by Hmmer, with full sequence E-value < 0.0001, full sequence score > 200 and bias < 0.1.

Additional File 5. Reference data.

Id and species of reference genomes from CARD database, resistance mechanism searched in CARD database and Outer groups for the phylogenetic analysis.

Additional File 6. Phylogenetic tree of aminoglycoside resistance genes.

Maximum-likelihood tree inferred with IQ-TREE v3.0.1 using the LG+F+G4 model. The tree includes 77 concatenated Salamanca sequences (220 amino acid sites, 219 informative) and homologous reference genes from the CARD database. Node support was evaluated with 1,000 ultrafast bootstrap replicates and 1,000 SH-aLRT tests.

Additional File 7. Phylogenetic tree of β-lactamase resistance genes.

Maximum-likelihood phylogeny based on 4,387 Salamanca sequences (204 amino acid sites, 204 informative) and CARD reference β-lactamases. Constructed with IQ-TREE (LG+F+G4 model), with 1,000 UFboot and SH-aLRT support tests.

Additional File 8. Phylogenetic tree of colistin resistance phosphoethanolamine transferases.

Maximum-likelihood tree of 752 Salamanca sequences (312 amino acid sites) and homologous CARD genes, inferred with IQ-TREE (LG+F+G4). Node support: 1,000 ultrafast bootstraps and 1,000 SH-aLRT replicates.

Additional File 9. Phylogenetic tree of fosfomycin transferase resistance genes.

Maximum-likelihood phylogeny including 213 Salamanca sequences (416 amino acid sites, 411 informative) and corresponding CARD reference genes. Inferred with IQ-TREE under the LG+F+G4 model with 1,000 UFboot and SH-aLRT tests.

Additional File 10. Phylogenetic tree of glycopeptide resistance genes.

Tree generated from 7,783 Salamanca sequences (97 amino acid sites). Constructed with IQ-TREE (LG+F+G4 model) including CARD reference homologs. Node support calculated using 1,000 ultrafast bootstrap and SH-aLRT replicates.

Additional File 11. Phylogenetic tree of macrolide phosphotransferase resistance genes.

Maximum-likelihood tree including 23 Salamanca sequences (282 amino acid sites, 271 informative) and CARD reference sequences. Inferred with IQ-TREE (LG+F+G4 model) and supported by 1,000 UFboot and SH-aLRT replicates.

Additional File 12. Phylogenetic tree of methicillin resistance genes.

Maximum-likelihood analysis of 40 Salamanca sequences (522 amino acid sites, 481 informative) and homologous CARD genes. Inferred with IQ-TREE (LG+F+G4 model); node support derived from 1,000 ultrafast bootstrap and SH-aLRT replicates.

Additional File 13. Phylogenetic tree of ribosomal RNA methyltransferase resistance genes.

Tree constructed from 350 Salamanca sequences (335 amino acid sites) and CARD references using IQ-TREE (LG+F+G4). Branch support estimated via 1,000 ultrafast bootstrap and 1,000 SH-aLRT tests.

Additional File 14. Phylogenetic tree of rifampicin resistance genes.

Maximum-likelihood tree of 62 Salamanca sequences (248 amino acid sites, 245 informative) and homologous CARD genes. Built with IQ-TREE (LG+F+G4 model) with 1,000 UFboot and SH-aLRT node support replicates.

Additional File 15. Phylogenetic tree of sulfonamides / DHPS resistance genes.

Maximum-likelihood tree of 62 Salamanca sequences (268 amino acid sites, 251 informative) and homologous CARD genes. Built with IQ-TREE (LG+F+G4 model) with 1,000 UFboot and SH-aLRT node support replicates.

Additional File 16. Phylogenetic tree of tetracyclines resistance genes.

Maximum-likelihood tree of 62 Salamanca sequences (326 amino acid sites, 325 informative) and homologous CARD genes. Built with IQ-TREE (LG+F+G4 model) with 1,000 UFboot and SH-aLRT node support replicates.

Additional File 17. Statistical_results.

Table 1. Differential Abundance of gene resistance families (Deseq2 results), Table 2. Alfa diversity of gene resistance families (Richness, Shannon and Simpson index), Table 3. Beta diversity of gene resistance families (Jaccard distances), Multivariate Permutational Analysis of Variance, Analysis of Variance Table, Multilevel pattern analysis.
