# Supplementary figures and images for: Metagenomic Comparison of Bat Colony Resistomes Across Anthropogenic and Pristine Habitats
Source: Antibiotics (Basel). 2026 Jan 3;15(1):51. doi: 10.3390/antibiotics15010051 (PMC12838372; doi:10.3390/antibiotics15010051)

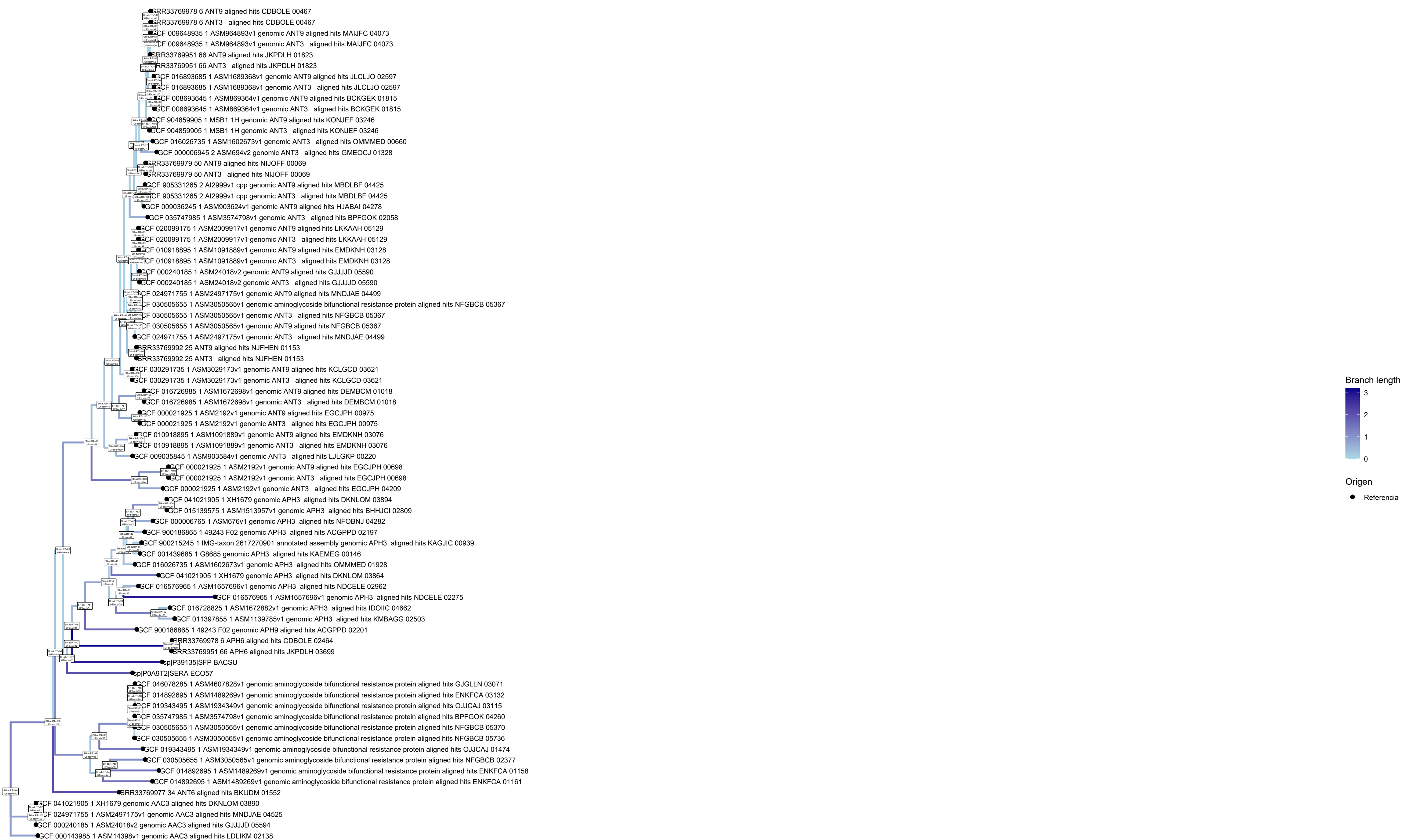

Supplement: Supplementary file 1 [file antibiotics-15-00051-s001.zip › Aminoglucosides.pdf]

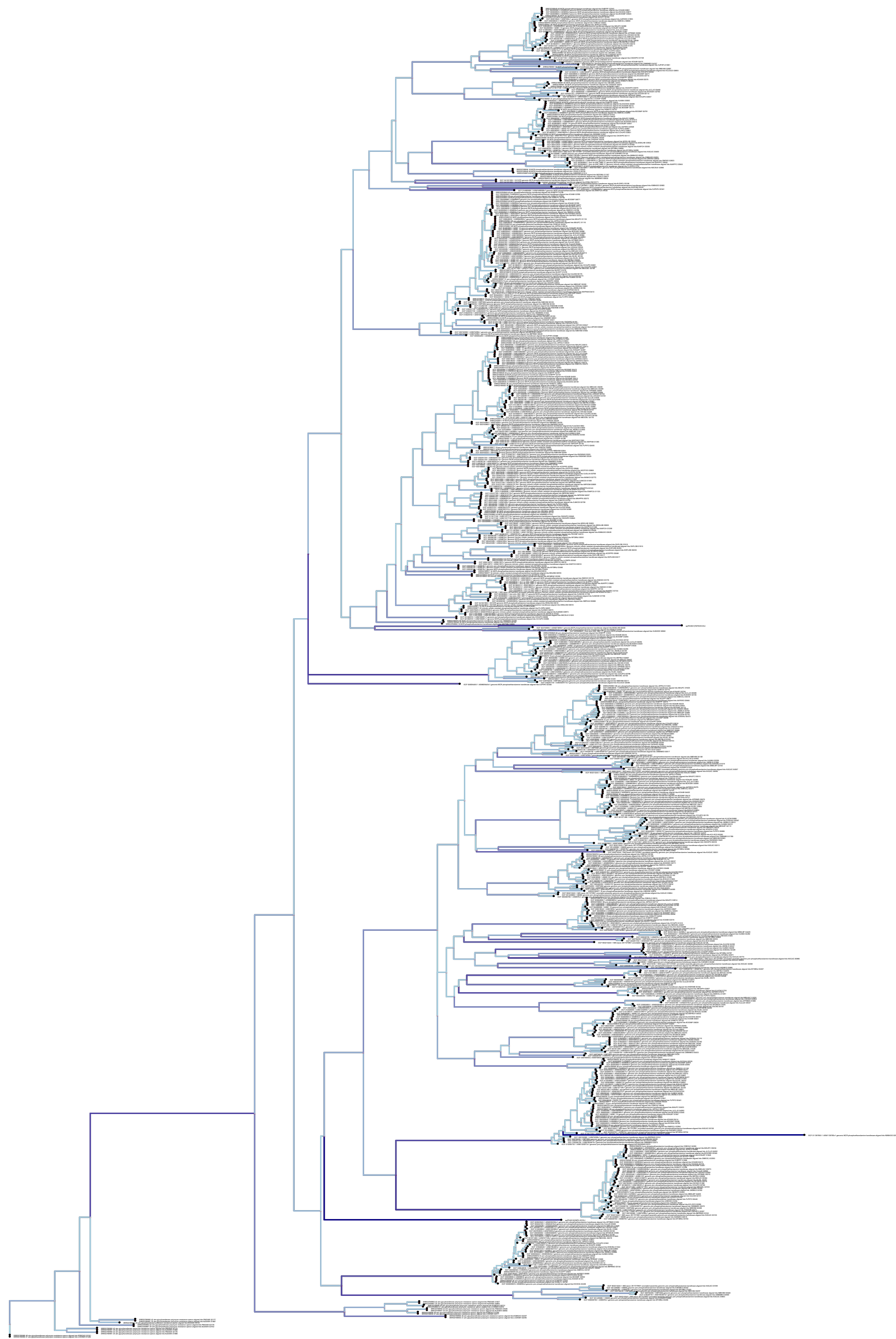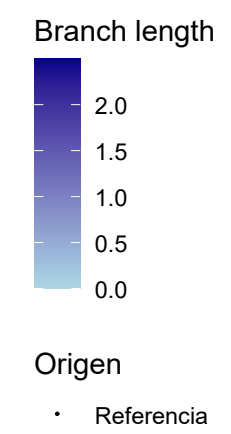

Supplement: Supplementary file 1 [file antibiotics-15-00051-s001.zip › Colistin _phosphoethanolamine_transferases.pdf]

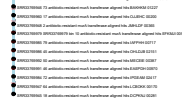

- Referencia

Supplement: Supplementary file 1 [file antibiotics-15-00051-s001.zip › Fosfomycin_transferases.pdf]

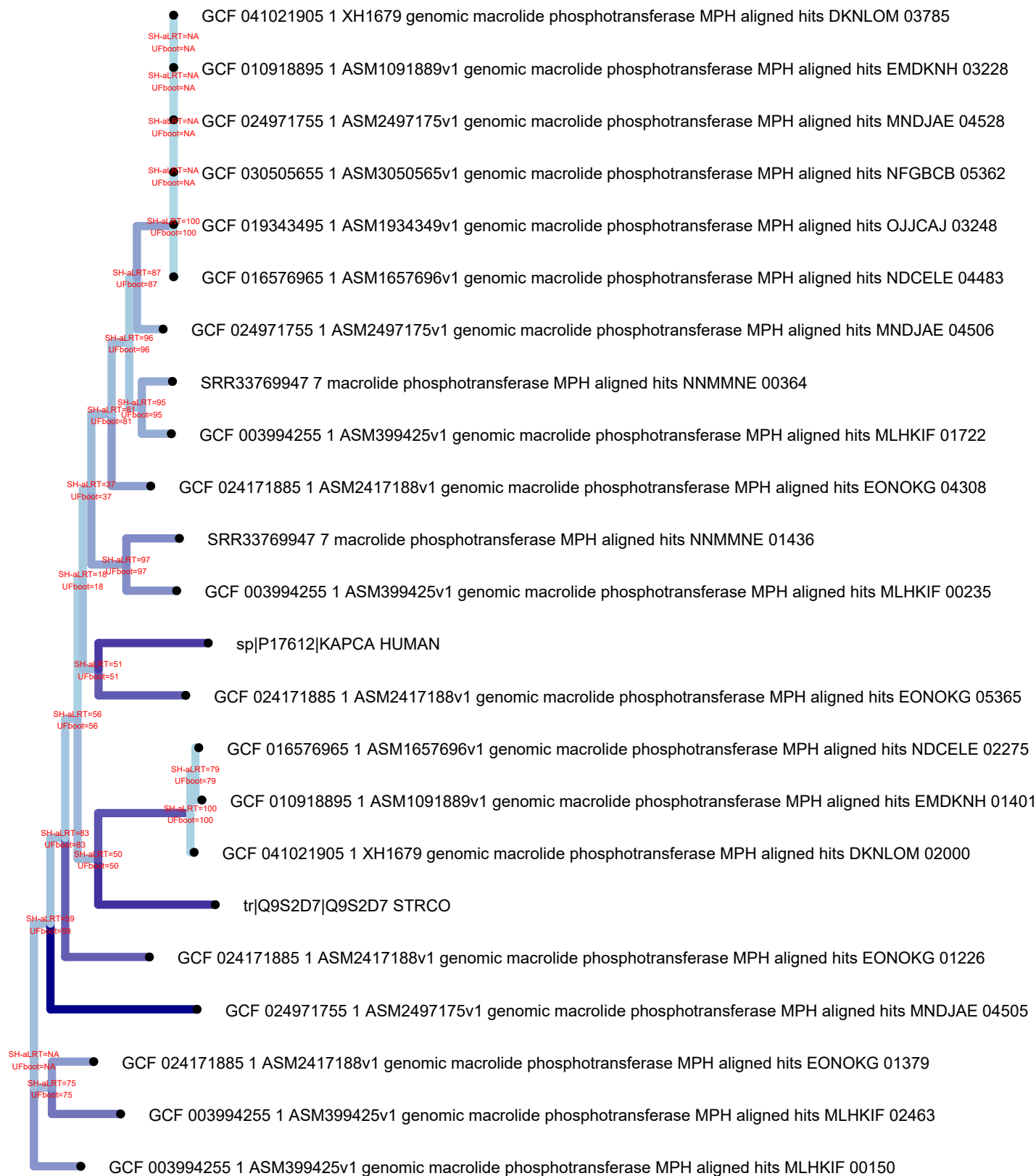

Branch length

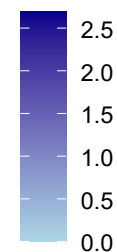

Origen

● Referencia

Supplement: Supplementary file 1 [file antibiotics-15-00051-s001.zip › Macrolide_phosphotransferase.pdf]

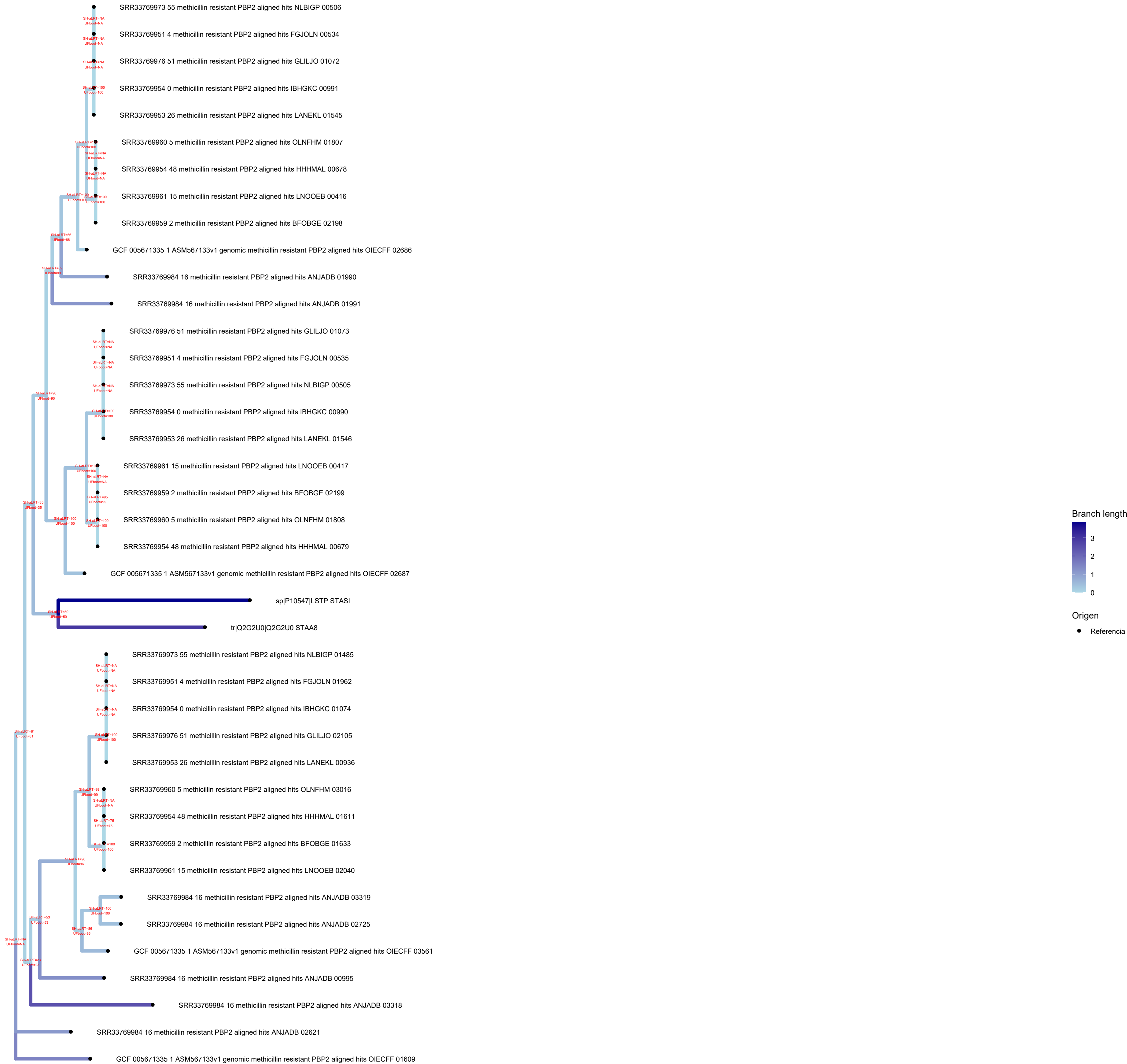

Supplement: Supplementary file 1 [file antibiotics-15-00051-s001.zip › Methicillin.pdf]

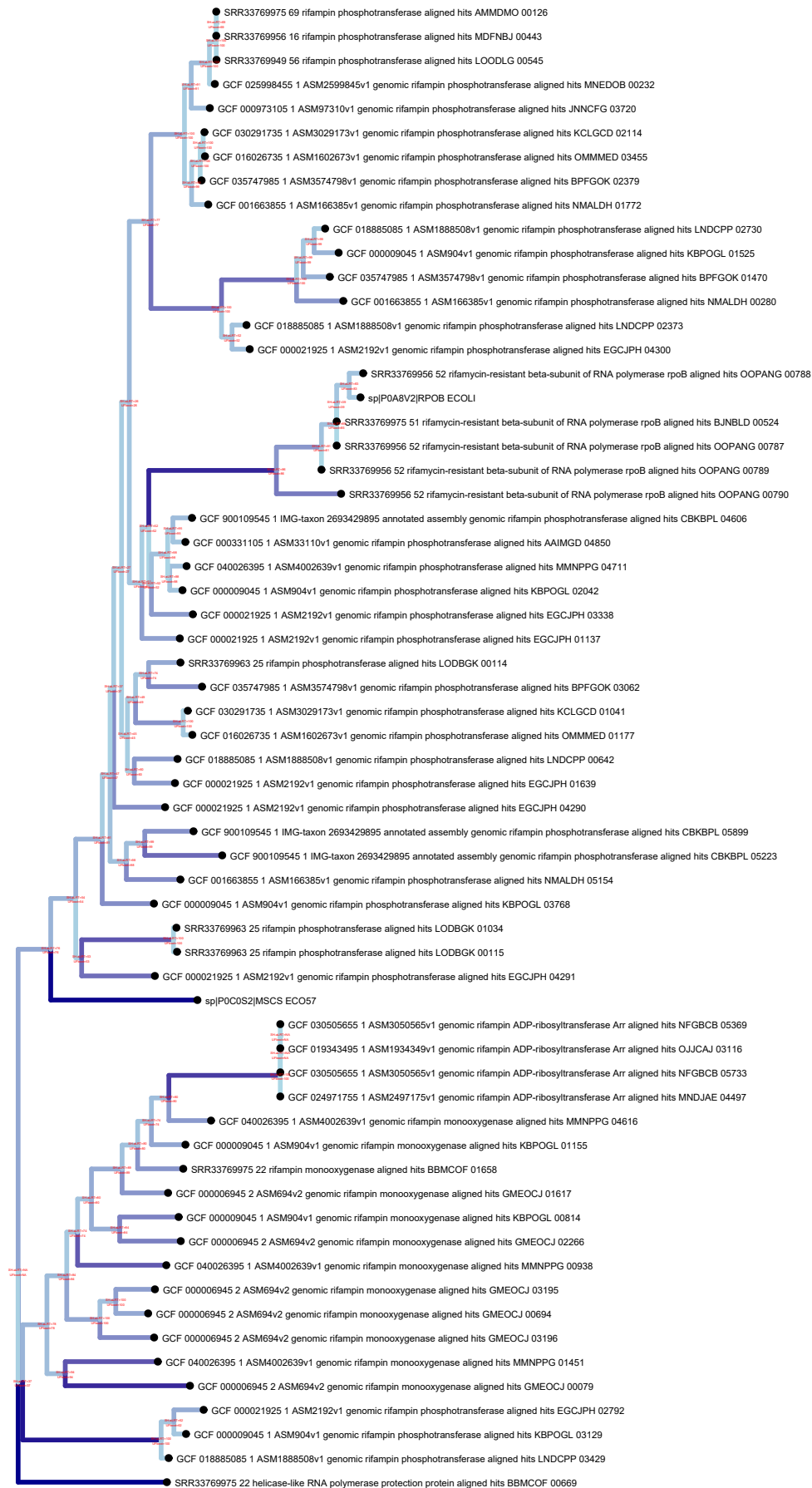

Branch length

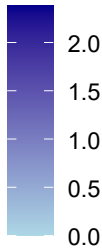

Origin

- Referencia

Supplement: Supplementary file 1 [file antibiotics-15-00051-s001.zip › Rifampicin.pdf]

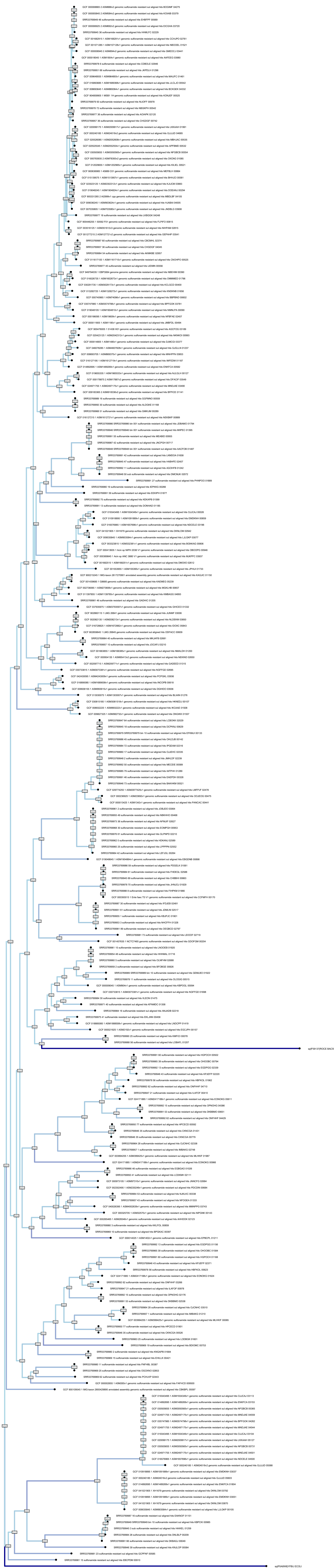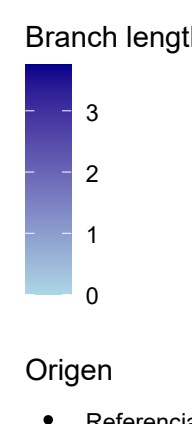

Supplement: Supplementary file 1 [file antibiotics-15-00051-s001.zip › Sulfonamides_DHPS.pdf]
