# Supplementary material for: Metagenomic Comparison of Bat Colony Resistomes Across Anthropogenic and Pristine Habitats
Source: Antibiotics (Basel). 2026 Jan 3;15(1):51. doi: 10.3390/antibiotics15010051 (PMC12838372; doi:10.3390/antibiotics15010051)
Supplement: Supplementary file 1 [file B-lactamases.pdf › B-lactamases.pdf]

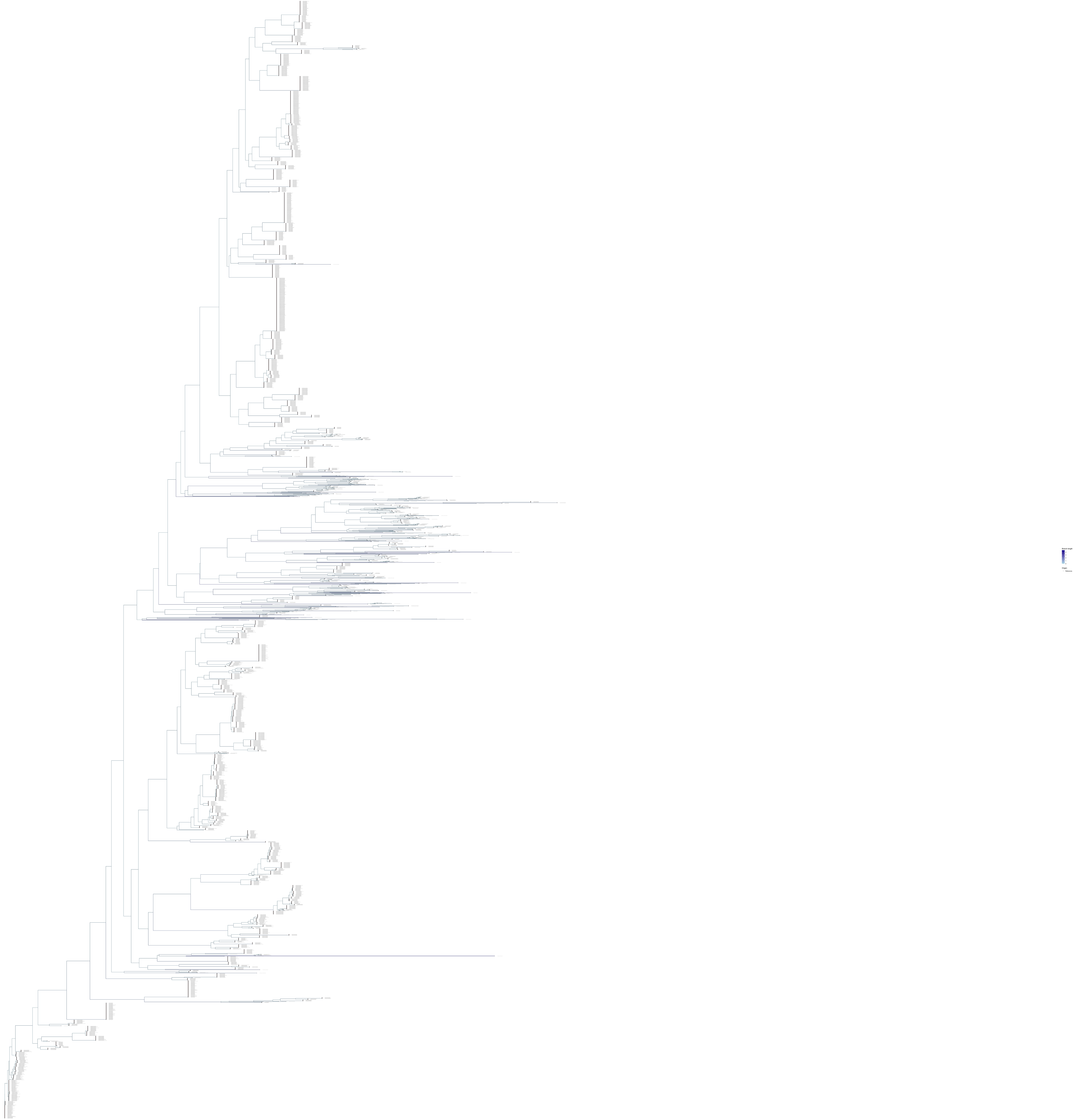
Branch length
4
3
2
1
0
Origen
Referencia
