# Supplementary material for: Metagenomic Comparison of Bat Colony Resistomes Across Anthropogenic and Pristine Habitats
Source: Antibiotics (Basel). 2026 Jan 3;15(1):51. doi: 10.3390/antibiotics15010051 (PMC12838372; doi:10.3390/antibiotics15010051)
Supplement: Supplementary file 1 [file Glicopeptides.pdf › Glicopeptides.pdf]

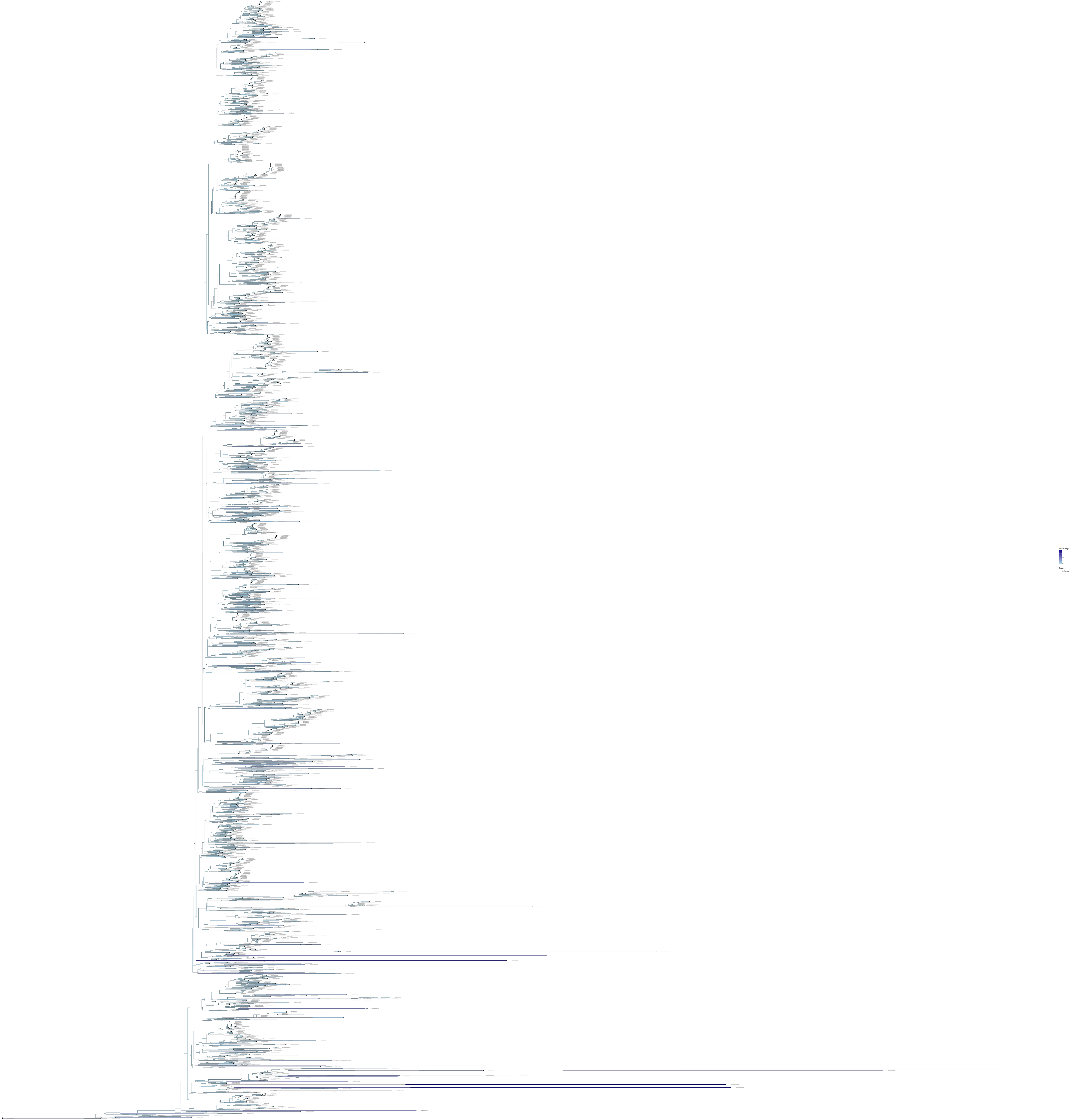

Branch length
7.5
5.0
2.5
0.0
Origen
Referencia
